# Tuning photoactivity and photoprotection of TiO_2_*via* TiO_2_/CeO_2_ heterostructure composite engineering

**DOI:** 10.1039/d6ra04759a

**Published:** 2026-07-28

**Authors:** Michał Gackowski, Dariusz T. Mlynarczyk, Halima Alem, Joanna Budna-Tukan, Tomasz Osmałek, Raphaël Schneider

**Affiliations:** a Université de Lorraine, CNRS, LRGP F-54000 Nancy France raphael.schneider@univ-lorraine.fr; b Poznan University of Medical Sciences, Chair and Department of Pharmaceutical Technology 3 Rokietnicka Street 60-806 Poznan Poland; c Poznan University of Medical Sciences, Doctoral School 70 Bukowska Street 60-812 Poznan Poland; d Poznan University of Medical Sciences, Chair and Department of Chemical Technology of Drugs Rokietnicka 3 60-806 Poznań Poland; e Université de Lorraine, CNRS, IJL F-54000 Nancy France; f Poznan University of Medical Sciences, Department of Immunology 60-806 Poznań Poland; g Department of Anatomy and Histology, Collegium Medicum, University of Zielona Gora 65-046 Zielona Gora Poland

## Abstract

Titanium dioxide (TiO_2_) is a widely used UV-shielding excipient for topical pharmaceutical and cosmetic formulations, but its ability to generate reactive oxygen species under UV irradiation limits its safety. Here, we report a MOF-derived TiO_2_/CeO_2_ heterostructure designed to suppress TiO_2_ photoactivity while preserving its UV-protective properties. Under UVA irradiation, CeO_2_ incorporation strongly inhibits superoxide radical generation and reduces hydroxyl radical formation by *ca.* 58% compared with rutile TiO_2_. Importantly, this reduced photoactivity translates into improved pharmaceutical performance: in hydrogel formulations, 0.1 wt% TiO_2_/CeO_2_ protects ketoprofen more effectively than TiO_2_ (77.54% *vs.* 52.56% remaining after irradiation), while 0.5 wt% TiO_2_/CeO_2_ provides almost complete photoprotection. The composite also has a lower impact on hydrogel matrix stability than pristine TiO_2_ and shows no acute toxicity in the Microtox assay. Overall, these results demonstrate that TiO_2_/CeO_2_ heterostructuring is an effective, safe-by-design strategy for developing photoprotective excipients that stabilize photosensitive drugs in topical formulations.

## Abstract 2

TiO_2_/CeO_2_ composites show UVA screening properties and markedly reduced ROS photoproduction.

## Graphical abstract

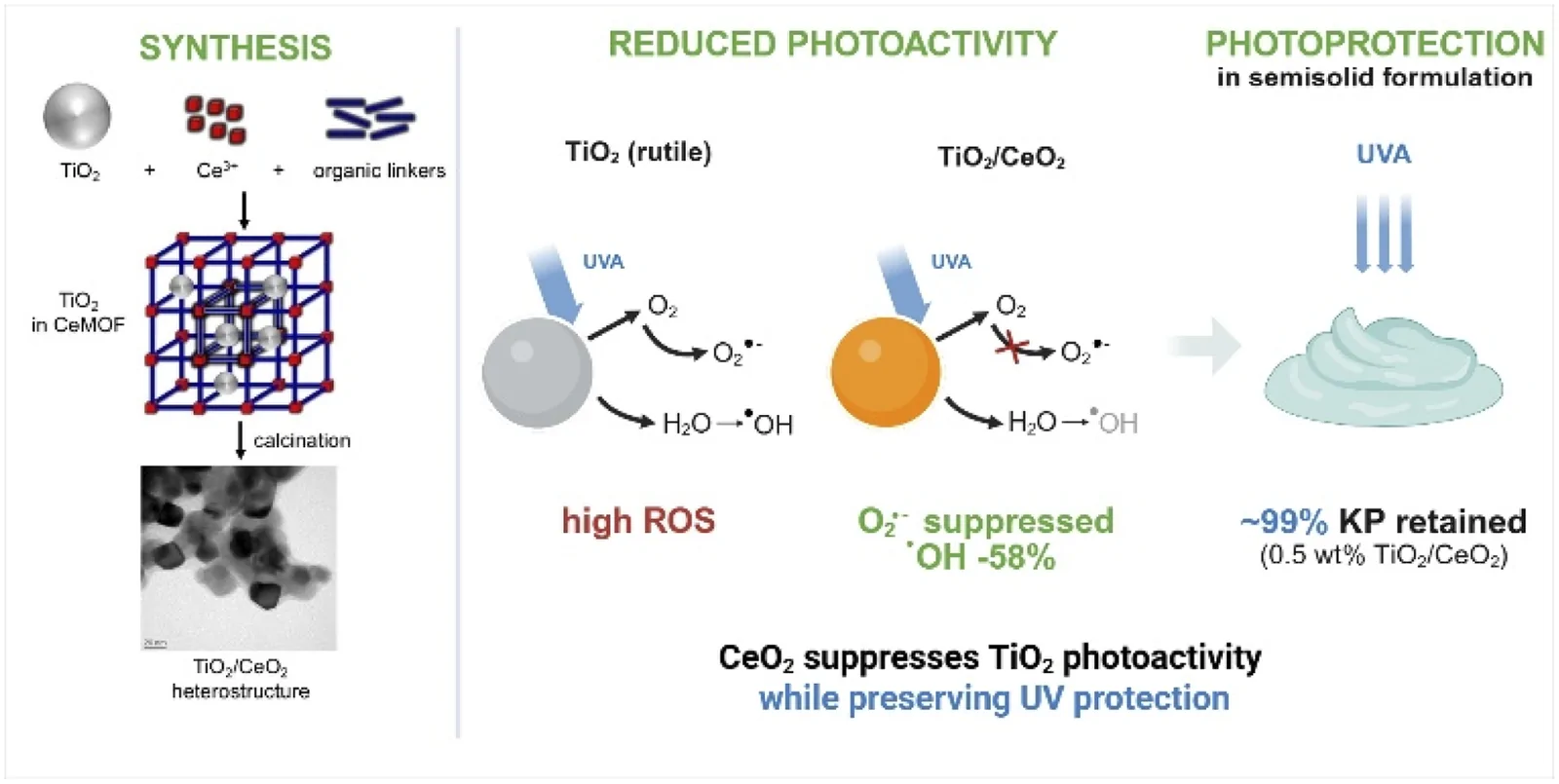

## Introduction

While solar radiation is essential in maintaining biological balance, it can also affect pharmacotherapy by causing photochemical degradation of drugs. Upon light exposure, particularly UV radiation, active pharmaceutical ingredients (APIs) may degrade and form reactive and potentially harmful photoproducts. This phenomenon, known as drug-induced photosensitization, is especially relevant in topically applied formulations. The scale of this issue is substantial as nearly 400 active substances have been identified as photosensitive.^1^ Drug-induced photosensitization may account for up to one-third of all skin-related adverse effects and for *ca.* 15% of photodermatoses.^2^ Moreover, retrospective data indicate that photochemical reactions constitute a measurable fraction of all reported adverse drug reactions, although their actual incidence is likely underestimated.^1,3^ Epidemiological observations further suggest an increasing trend in the prescription of potentially photosensitizing medications.

From a mechanistic perspective, photosensitization involves the activation by light of a compound followed by reactions including photodegradation, loss of pharmacological activity, and generation of reactive photoproducts able to induce adverse biological effects. In dermatological pharmacotherapy, drugs such as analgesics,^4^ antibiotics,^5^ antiallergics,^6^ and anti-acne agents^7^ are applied directly to the skin. Among light sources, UVA radiation is considered the most deleterious since it penetrates deeper into the skin and initiates photochemical reactions.^8^ The effects of photosensitization may vary from mild erythema and irritation to more severe skin damage. Importantly, the occurrence of these reactions often requires the therapy to be discontinued, potentially compromising treatment efficacy and requiring alternative therapeutic strategies.^9^ Therefore, there is a growing need for the development of formulations able to enhance the photostability of APIs while minimizing the risk of light-induced degradation.

One strategy to address drug photosensitivity involves using excipients that absorb, scatter, or reflect radiations, thereby limiting light penetration and reducing its impact on skin. Inorganic UV filters like TiO_2_ have attracted particular attention due to their stability and broad light absorption and are therefore used in pharmaceutical and cosmetic formulations.^10^ Moreover, TiO_2_ light-scattering properties allow the protection of APIs from light-induced degradation.^8,11,12^ However, TiO_2_ is also a photocatalyst and may generate harmful reactive oxygen species (ROS) like hydroxyl and superoxide radicals which may contribute to oxidative degradation of APIs and potentially enhance phototoxic effects.^13^ Recent research has thus focused on modifying TiO_2_ to suppress its photoactivity while preserving its UV-blocking efficiency, thereby improving its suitability in pharmaceutical formulations. Concerning the safety of TiO_2_, concerns raised in 2021 when the European Medicines Agency recommended the search for alternatives to TiO_2_ in formulations due to its potential genotoxicity.^14^ The use of TiO_2_ as a food additive has been restricted in the European Union while the FDA still classifies TiO_2_ as safe and effective (GRASE, Category I).^15^ This highlights the need to develop new or modified materials with improved safety characteristics. Numerous strategies have been developed to mitigate the phototoxicity of TiO_2_ by modifying its surface properties, for example core/shell structures using inert coatings such as silica (SiO_2_), alumina (Al_2_O_3_), or siloxanes^16^ to reduce TiO_2_ photocatalytic activity while preserving its UV-shielding properties. Although such strategies proved to be efficient,^17–20^ complete inhibition of ROS photoproduction remains challenging.^16,21^ Moreover, the introduced coatings may alter the interactions of nanoparticles (NPs) with biological systems, raising additional concerns about their safety and potential toxicity.^22–24^

Among coatings, CeO_2_ has emerged as a promising material to tune the photoactivity of TiO_2_. The deposition of CeO_2_ on the surface of TiO_2_ reduces ROS production while extending the UV-vis absorption.^25,26^ Similar effects have been reported for oleyl phosphate-modified TiO_2_/Ce_2_O_3_ nanoparticles, indicating that interfacial engineering may also be used to control photoactivity.^27^

The unique redox properties of ceria (reversible switch between Ce^3+^ and Ce^4+^ oxidation states) allow CeO_2_ to exhibit enzyme-mimetic behavior and to scavenge ROS. CeO_2_ was demonstrated to promote charge-carrier recombination or to act as a radical scavenger.^28^ In addition, CeO_2_ shows also protective biological activity and its redox-active surface mitigates oxidative stress and thus may enhance repair processes and preserve cell viability under UV exposure.^29^ Furthermore, CeO_2_ is also able to trap hydroxyl radicals^29^ and thus counteracts the harmful effects of TiO_2_-induced photoactivity. Additionally, CeO_2_ shows a favorable safety profile, further supporting its use in pharmaceutical applications.^28^

The TiO_2_–CeO_2_ interface was demonstrated to play a key role on the photoactivity.^30^ Thus, controlling the interfacial organization between both oxides represents an effective strategy to modulate the photoactivity while preserving UV-shielding properties. In this context, a MOF-based synthetic route was selected as a rational platform to pre-organize the ceria precursor around TiO_2_ prior to thermal treatment, thereby facilitating the formation of TiO_2_/CeO_2_ interfacial contact in the final composite.

Herein, we report the preparation of TiO_2_/CeO_2_ composites with reduced photoactivity while maintaining photoprotective properties. Under UVA illumination, the new materials engineered effectively protect drugs like ketoprofen (KP) both in aqueous solution and pharmaceutical formulations like hydrogels.

## Experimental

### Materials

Ammonium cerium nitrate (NH_4_)_2_Ce(NO_3_)_6_ (Fischer, 99%), titanium dioxide TiO_2_ rutile nanopowder (<100 nm, Merck, 99.5%), terephthalic acid H_2_BDC (Fischer, 99+%), disodium terephthalate DST (Fischer, 99+%), *N*,*N*-dimethylformamide DMF (Merck, ≥99.8%), acetic acid (glacial, VWR, ACS reagent), triethylamine TEA (Fischer, 99%), acetone (VWR), ethanol absolute (VWR), acetonitrile (VWR, HPLC grade), potassium phosphate monobasic KH_2_PO_4_ (Fischer, 99+%), hydrochloric acid (Merck, 37%), sodium hydroxide (VWR, ≥99%), nitroblue tetrazolium chloride NBT (Merck, 98%), dimethylsulfoxide DMSO (Merck, ≥99.5%), ethylenediaminetetraacetic acid EDTA (Merck, analytical reagent), 4-hydroxy-2,2,6,6-tetramethylpiperidine 1-oxyl (TEMPOL, Fischer, 99+%), hydrochloric acid (Merck, 37%), sodium hydroxide (VWR, ≥99%), *tert*-butanol (VWR, 99.5%), ketoprofen KP (Pol-Aura, 98%), crystal violet CV (Fischer), ≥96%), Carbopol® EZ-3 polymer (Lubrizol), and triisopropanolamine TIPA (Fischer, 98%) were used as received. All aqueous solutions were prepared with ultrapure water of 18.2 MΩ cm resistivity.

### Synthesis of TiO_2_/CeO_2_ composite

**Fig. 1:**
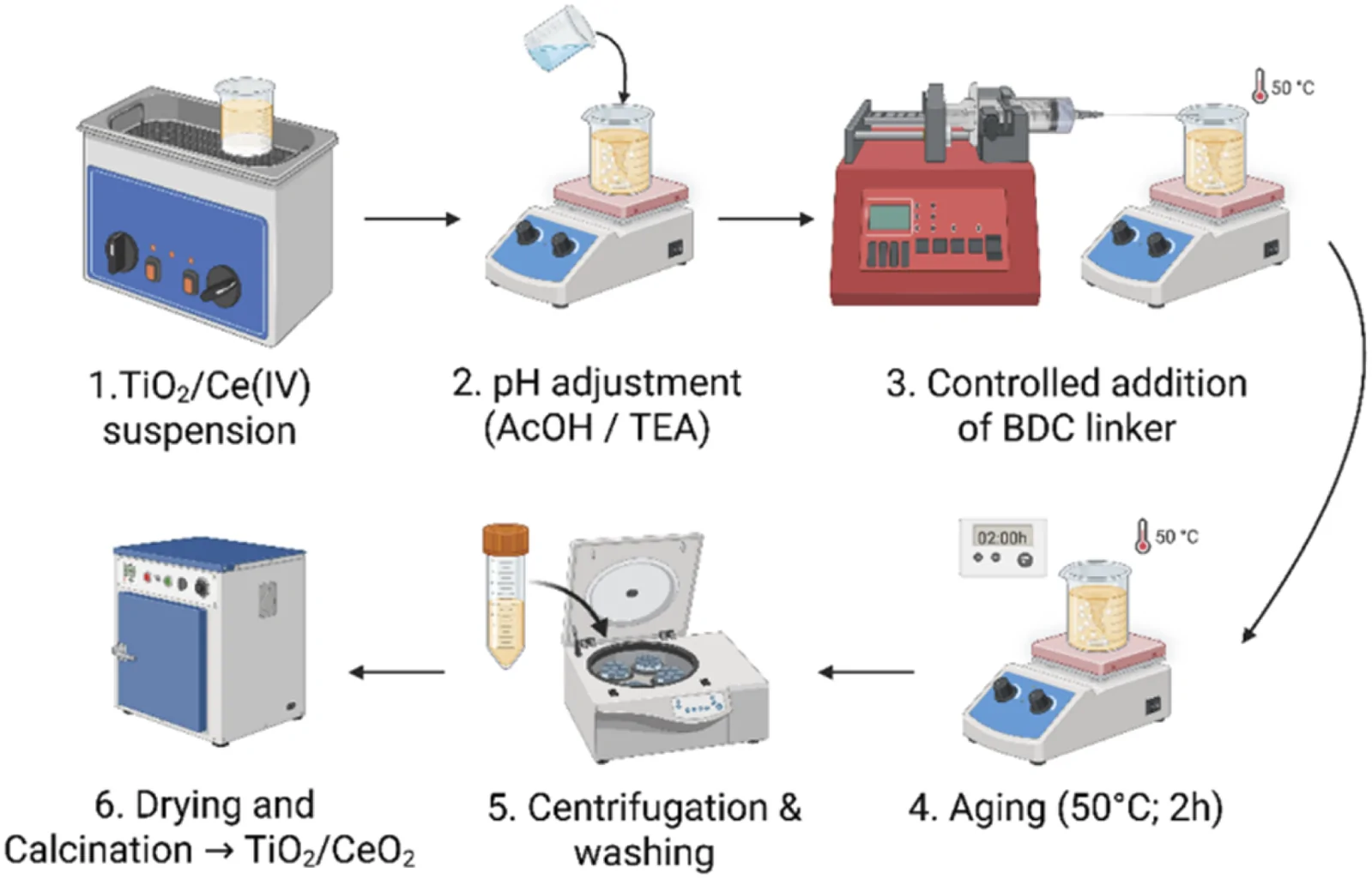
Schematic representation of the synthesis of composite TiO_2_/CeO_2_ nanoparticles.

The synthesis of the cerium-based metal–organic framework was adapted from a previously reported procedure for Ce-UiO-66-H with significant modifications.^31^ The synthesis procedure is schematically illustrated in Fig. 1. (NH_4_)_2_Ce(NO_3_)_6_ (0.3 g) was dissolved in 3 mL of H_2_O. Subsequently, TiO_2_ (0.078 g) was added, and the mixture was sonicated for 15 min to obtain a homogeneous suspension (solution 1). In a separate beaker, 10 mL of H_2_O was heated to 53 °C, followed by the addition of CH_3_COOH (2 mL) and TEA (18 drops), yielding solution 2. Solutions 1 and 2 were then combined and stirred at 50 °C for 30 min.

Meanwhile, solution 3 was prepared by dissolving H_2_BDC (0.3 g) in 30 mL of DMF. After 30 min of mixing solutions 1 and 2, solution 3 was added dropwise to the reaction mixture using a syringe with a needle at a rate of one drop every 5 s while maintaining the temperature at 50 °C. After complete addition, the resulting suspension was maintained at 50 °C for 2 h.

The obtained mixture was centrifuged (5000 rpm, 5 min), and the precipitate was collected. The solid was washed three times with acetone (15 mL) and then with absolute ethanol (15 mL). The product was then dried at 110 °C for 1.5 h, yielding the TiO_2_/UiO-66(Ce) hybrid.

For further studies, the obtained material was calcined under various conditions (400–800 °C, heating rates of 30–50 °C min^−1^, and durations of 2–4 h). Based on these experiments, the optimal calcination conditions were determined to be 800 °C, with a heating rate of 30 °C min^−1^ and a duration of 2 h, resulting in the final TiO_2_/CeO_2_ composite.

### Physicochemical characterizations

X-ray diffraction (XRD) patterns were recorded using a PANalytical X'Pert Pro MPD and Rigaku SmartLab diffractometers with Cu Kα radiation (*λ* = 0.15406 nm). Thermogravimetric analysis (TGA) and Differential Scanning Calorimetry (DSC) were performed using a TGA/DSC1 STAR system (Mettler-Toledo) in the temperature range of 30–800 °C with a heating rate of 10 °C min^−1^. Fourier transform infrared (FT-IR) spectra were recorded at room temperature in the range of 4000–360 cm^−1^ using a Bruker ALPHA II spectrometer. Scanning electron microscopy (SEM) images were obtained using a FlexSEM 1000 II microscope (Hitachi). Elemental composition and distribution were analyzed by two-dimensional elemental mapping using energy-dispersive X-ray spectroscopy (EDX). Transmission electron microscopy (TEM) was carried out using a JEOL JEM-ARM200F Cold FEG microscope operated at an accelerating voltage of 200 kV and equipped with a spherical aberration (Cs) probe corrector. Scanning transmission electron microscopy (STEM) combined with high-angle annular dark-field (HAADF) imaging and energy-dispersive X-ray spectroscopy (EDS) was used to investigate the elemental distribution. Specific surface area and pore size distribution were determined by nitrogen adsorption at 77 K using the Brunauer–Emmett–Teller (BET) method on a Micromeritics TRISTAR II Plus instrument. The zeta potential was measured using a Malvern Zetasizer Nano ZS to determine the material's isoelectric point. Diffuse reflectance spectra (DRS) were recorded using a Shimadzu UV-2600–2700 spectrophotometer. UV-visible absorption spectra were recorded on a Thermo Scientific Evolution 220 spectrometer. Photoluminescence (PL) emission spectra were recorded on a Fluoromax Horiba equipment. X-ray photoelectron spectroscopy (XPS) measurements were conducted using a Omicron EA125 analyser and a Al Kα excitation source (1486.6 eV).

### ROS generation studies

All experiments involving light irradiation were carried out using three UVA blacklight blue lamps (Philips TL-D 15W BLB) with a maximum emission at 365 nm. The light intensity at the sample position was measured using an Optical Power and Energy Meter PM100D (Thorlabs Instrumentation) at *λ* = 365 nm and was adjusted to 4 mW cm^−2^.

### Superoxide radical (O_2_˙^−^) generation

The generation of superoxide radicals (O_2_˙^−^) was evaluated using the nitroblue tetrazolium (NBT) assay, as described previously with minor modifications.^32,33^ Briefly, the method is based on the spectrophotometric detection of NBT reduction from a yellow compound to purple formazan derivatives. The formation of mono- and diformazan species results in an increase in absorbance over 450–700 nm. In a typical experiment, TiO_2_ or TiO_2_/CeO_2_ (2 mg) and NBT (8 mg) were dispersed in 100 mL of a water/DMSO mixture (1 : 1, v/v) under light-protected conditions. The suspension was then exposed to UVA irradiation for 90 min, and aliquots were collected at predetermined time intervals (0, 5, 10, 15, 30, 45, 60, and 90 min) and UV-visible absorption spectra were recorded. Control experiments included (i) NBT solution irradiated in the absence of the materials and (ii) NBT in the presence of the materials without light exposure. The results were expressed as the increase in absorbance at 650 nm, corresponding to the formation of formazan derivatives.

### Hydroxyl radical (˙OH) generation

The generation of hydroxyl radical (˙OH) was evaluated using the disodium terephthalate (DST) assay.^33^ This method is based on the reaction of ^˙^OH radicals with DST to form the fluorescent 2-hydroxyterephthalate (2-OH-DST), which exhibits an emission maximum at 428 nm. Briefly, TiO_2_ or TiO_2_/CeO_2_ particles (5 mg) were dispersed in 100 mL of H_2_O. Subsequently, 20 mL of the suspension was mixed with 20 mL of DST solution (0.1 M in water) and irradiated with UVA. Aliquots were collected at defined time intervals (0, 5, 10, 20, 30, 45, and 60 min). At each time point, 1 mL of the sample was withdrawn and mixed with 0.5 mL of NaOH solution (1 M), followed by incubation for 50 min at room temperature. The suspension was then centrifuged (15 000 rpm, 5 min) to remove the nanoparticles. The formation of 2-OH-DST was quantified by recording fluorescence spectra (*λ*_ex_ = 300 nm, *λ*_em_ = 428 nm) using a Horiba Fluoromax spectrometer equipped with a Xenon lamp as the excitation source.

### ROS scavenging experiments

Radical scavenging experiments were performed to identify the main reactive species involved in the photocatalytic degradation of KP. For this purpose, 4-hydroxy-TEMPO, *tert-*butanol, and ethylenediaminetetraacetic acid (EDTA) were used as scavengers for superoxide radical (O_2_˙^−^), hydroxyl radical (˙OH) and photogenerated holes (h^+^), respectively. Each scavenger was added at a concentration of 10 mM immediately prior to irradiation.

## KP degradation studies

### Quantification of KP by HPLC-UV

The quantification of KP was performed using a high-performance liquid chromatography system (LC-20AT Prominence, Shimadzu) equipped with a UV-vis detector (SPD-20A Prominence, Shimadzu). Manual sample injection was carried out using a 50 µL loop. Data acquisition and processing were performed using Galaxie Chromatography Data System software (version 1.9.305.530, Varian). Chromatographic separation was achieved on a Merck KGaA LiChrosorb® RP-18 column (5 µm, 250 mm × 4.6 mm). The mobile phase consisted of acetonitrile and 0.02 M KH_2_PO_4_ aqueous solution (60 : 40, v/v), adjusted to pH 2.5 with acetic acid. The flow rate was set to 0.5 mL min^−1^ and detection was performed at 254 nm. The method was validated for specificity, linearity, limit of detection (LOD), limit of quantification (LOQ), precision, and accuracy. Linearity was evaluated in the concentration range of 0.1–15 µg mL^−1^. The LOD and LOQ were determined as 0.581 ± 0.148 µg mL^−1^ and 1.759 ± 0.449 µg mL^−1^, respectively.

Accuracy was equal to 3.9% at a low concentration level (1.12 µg mL^−1^). Precision, expressed as the coefficient of variation, was equal to 1.34%.

### Photodegradation of KP in hydrogel

The photodegradation of KP in hydrogels was carried out under UVA irradiation. The composition of the investigated hydrogels is presented in Table S1.

Hydrogels were prepared by dissolving KP in a mixture of ethanol and water, then adding Carbopol® EZ-3 under magnetic stirring until a homogeneous suspension was obtained. Subsequently, TIPA, previously dissolved in 0.2 mL of water, was added to neutralize and initiate gel formation. The mixture was further homogenized using manual stirring until a uniform gel was formed. When applicable, TiO_2_ or TiO_2_/CeO_2_ was incorporated into the formulation and mixed until a homogeneous distribution within the hydrogel matrix was achieved. All hydrogels were prepared at room temperature.

The hydrogels were placed in a quartz-glass cuvette with a 4 mm layer thickness. The cuvette was sealed with a lid to prevent solvent evaporation. The samples were irradiated for 240 min, and hydrogel samples were collected at defined time intervals (0, 30, 60, 120, 180, and 240 min). At each time point, 50 mg of hydrogel was withdrawn and extracted using 10 mL of absolute ethanol under ultrasonic treatment. The obtained suspension was centrifuged (15 000 rpm, 15 min) and 0.1 mL of the supernatant was collected and diluted with 0.4 mL of the mobile phase prior to analysis. KP concentration was determined by HPLC-UV as described previously and the results were expressed as the normalized concentration ratio (*C*_*t*_/*C*_0_).

For comparative purposes, the photostability enhancement factor (PEF) was calculated for the investigated formulations and defined as the ratio of KP degradation in the blank hydrogel to the degradation observed in the corresponding tested formulation after the selected irradiation time:PEF = *D*_blank_/*D*_sample_where *D*_blank_ is the percentage of KP degraded in the hydrogel without added material at a specific time, and *D*_sample_ is the percentage of KP degraded in the formulation containing the tested material at a specific time.

### UV-vis spectroscopy of hydrogel extracts

The stability of the hydrogel matrix under UVA irradiation was evaluated using hydrogels prepared as described previously, but without KP. Blank hydrogels (without inorganic material) as well as hydrogels containing 0.1% of TiO_2_ or TiO_2_/CeO_2_ were investigated. The samples were exposed to UVA irradiation for 180 min under the conditions described previously. At defined time intervals, hydrogel samples were collected for analysis. For each time point, 50 mg of hydrogel was dissolved in 1.5 mL of an ethanol–water mixture (30.69 : 69.31, v/v). The same solvent mixture was used as a baseline for spectroscopic measurements. UV-vis absorption spectra were recorded over 190–800 nm.

### Ecotoxicity assessment – microtox acute toxicity study

Acute toxicity was evaluated using a Microtox® M500 analyzer (Modern Water plc, London, United Kingdom) operated with Microtox Omni 4.2 software (Modern Water plc, London, United Kingdom). All instrumentation, reagents, and consumables required for the assay were supplied by Tigret Sp. z o.o. (Warsaw, Poland). Liquid samples were analyzed according to the 81.9% screening protocol described in ISO 11348-3:2007. The Microtox acute reagent, consisting of lyophilized *Aliivibrio fischeri*, was reconstituted at 5 °C using the manufacturer-provided reconstitution solution (ultrapure water) and subsequently diluted tenfold with Microtox diluent (2% NaCl solution). The bacterial suspension was equilibrated at 15 °C for 15 min prior to measurements. The baseline bioluminescence of the bacterial suspension was recorded, followed by the immediate addition of the tested sample, which had been premixed with osmotic adjusting solution (22% NaCl) and pre-equilibrated to 15 °C. The inhibition of bioluminescence was determined after 5 and 15 min of exposure. A control measurement was performed using Microtox diluent under identical conditions.

Suspensions of TiO_2_, CeO_2_, and TiO_2_/CeO_2_ were tested at four different concentrations (0.1%, 0.2%, 0.5%, and 1.0%, w/v).

## Results and discuss

### Material synthesis and characterizations

The study was initiated by optimizing the TiO_2_/CeO_2_ composition prior studying the effect of calcination conditions. Three different compositions, namely TiO_2_/CeO_2_ (1 : 0.6, 1 : 1.2, and 1 : 1.8, w/w) were screened by evaluating the photocatalytic degradation of Crystal Violet (CV) using TiO_2_/CeO_2_ composites under UVA irradiation. Results show that TiO_2_/CeO_2_ (1 : 1.2) exhibited the lowest activity for CV degradation and was therefore selected for all subsequent studies, including calcination optimization, mechanistic investigations, and photoprotection experiments.

**Fig. 2:**
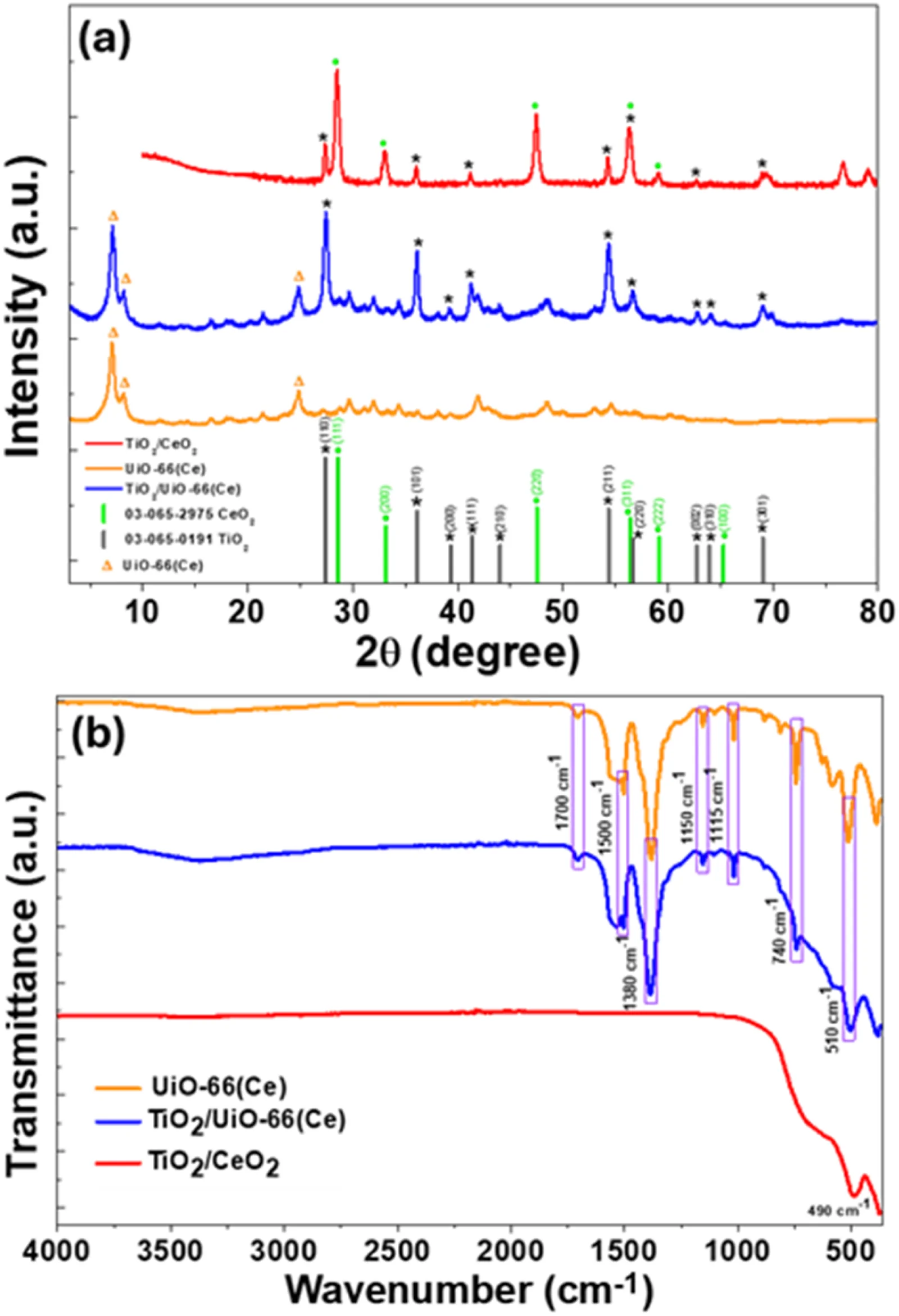
(a) XRD patterns and (b) FT-IR spectra of UiO-66(Ce), TiO_2_/UiO-66(Ce) precursor and calcined TiO_2_/CeO_2_ heterostructure. Reference patterns corresponding to CeO_2_ (PDF 03-065-2975) and TiO_2_ (PDF 03-065-0191) are shown for comparison.

Using the optimized composition, a TiO_2_/UiO-66(Ce) hybrid material was prepared *via in situ* growth of the UiO-66-Ce MOF in the presence of TiO_2_ NPs. Powder XRD confirmed the successful formation of the UiO-66-type cerium framework, as evidenced by the characteristic reflections of the face-centered cubic phase peaks located at 2*θ* values of 7.1 and 8.2°, together with the diffraction features of TiO_2_ (Fig. 2a). These results indicate the successful preparation of the TiO_2_/UiO-66(Ce) precursor. CeO_2_ obtained after calcination of UiO-66(Ce) exhibits a cubic structure with the space group *Fm*3̄*m* (PDF 03-065-2975).

**Fig. 3:**
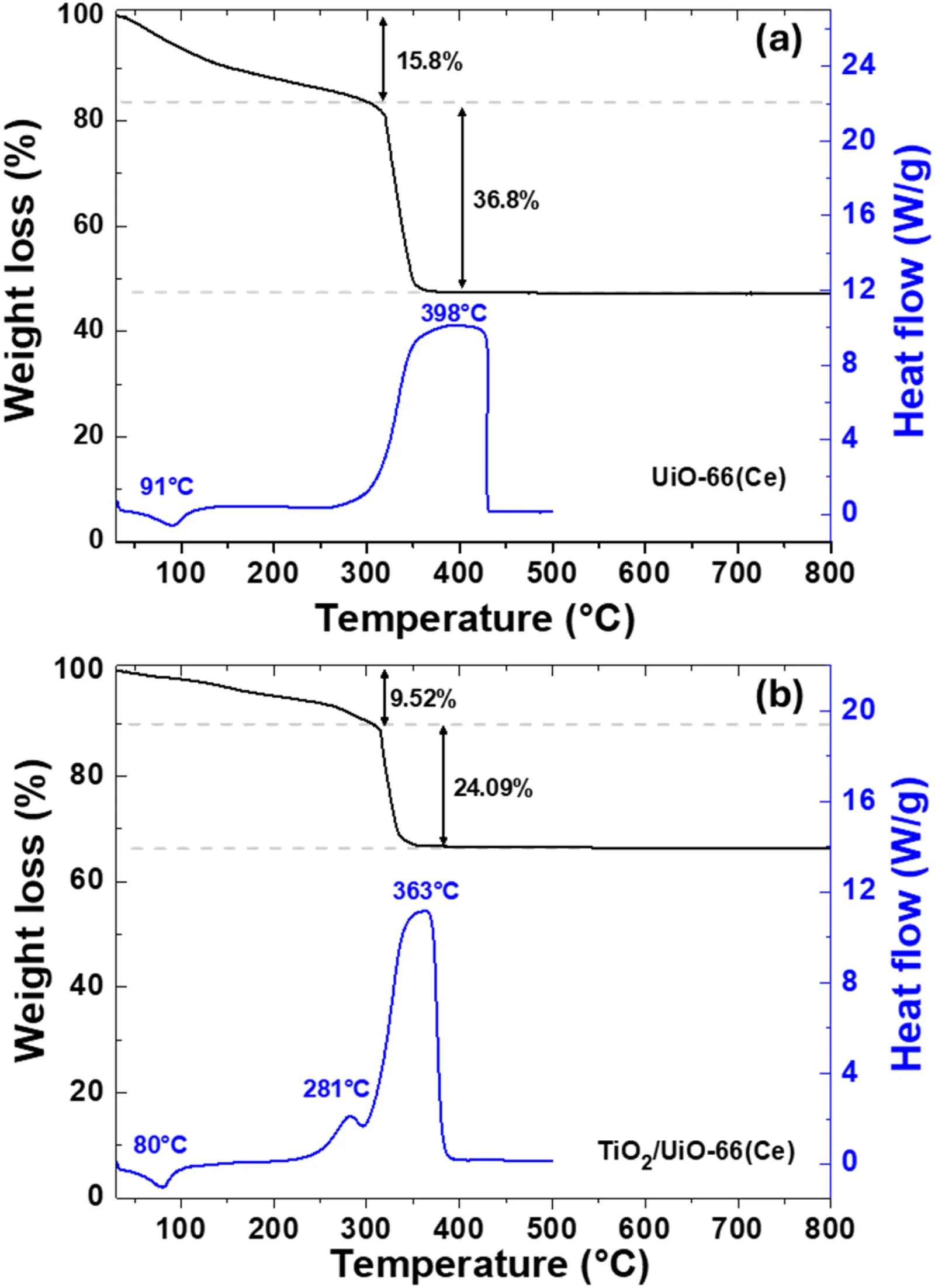
TGA and DSC curves of (a) UiO-66(Ce) and (b) TiO_2_/UiO-66(Ce) precursor recorded under air atmosphere.

FT-IR spectroscopy was used to monitor the structural evolution during precursor calcination (Fig. 2b). The spectrum of UiO-66(Ce) exhibits characteristic signals associated with the BDC linker and the Ce inorganic node. The band observed at 1700 cm^−1^ can be assigned to C=O stretching vibrations of residual non-coordinated carboxylic groups, while the intense bands at 1500 and 1380 cm^−1^ correspond to the asymmetric and symmetric stretching modes of coordinated carboxylate groups (COO^−^), respectively, which are characteristic of UiO-66 framework.^34^ Additional bands located at 1150 and 1115 cm^−1^ may be attributed to C–O and aromatic ring vibrations, whereas the signal at 740 cm^−1^ may originate from out-of-plane C–H bending of the *para*-disubstituted benzene ring. The low-frequency signal centered at 510 cm^−1^ was assigned to Ce–O stretchings within the metal cluster.^34^ The FT-IR spectrum of the TiO_2_/UiO-66(Ce) precursor retained the characteristic linker-related bands at 1500 and 1380 cm^−1^, confirming preservation of the UiO-66(Ce) framework after incorporation of TiO_2_ NPs. At the same time, signals in the low-wavenumber region indicated contributions from Ti–O–Ti and Ti–O vibrations overlapping with Ce–O modes. FT-IR and XRD results confirm the successful formation of the TiO_2_/UiO-66(Ce) precursor^35^ through *in situ* growth of the UiO-66(Ce) phase in the presence of TiO_2_. After calcination, the disappearance of the characteristic signals in the 1700–1000 cm^−1^ region confirmed complete decomposition of the BDC linker and collapse of the MOF framework. The resulting spectrum was dominated by broad metal–oxygen stretchings below 700 cm^−1^, with a prominent band centered at 490 cm^−1^ assigned to Ti–O–Ti, Ti–O, and Ce–O lattice vibrations. The shift of the Ce–O signal from 510 cm^−1^ in UiO-66(Ce) to 490 cm^−1^ in the calcined sample may be attributed to the change in Ce coordination environment, from Ce–carboxylate to Ce–O bonds.^34^ TGA and DSC were performed for both UiO-66(Ce) and the TiO_2_/UiO-66(Ce) precursor in order to determine the thermal stability of the materials and identify the minimal temperature required for complete decomposition of the MOF into CeO_2_ (Fig. 3). The endothermic peaks observed at 91 and 80 °C for UiO-66(Ce) and UiO-66(Ce)/TiO_2_, respectively, likely correspond to the removal of gaseous molecules adsorbed in the UiO-66(Ce) framework. Then, up to *ca.* 300 °C, the gradual weight loss observed for both materials can be attributed to the removal of crystallization water, residual solvents, and volatile species trapped within the MOF porous structure. For UiO-66(Ce), the weight loss was 15.8%, whereas for the TiO_2_/UiO-66(Ce) precursor, it was 9.52%. The relatively smooth and continuous decrease in mass suggests that the post-synthetic purification and washing procedure were effective. The decomposition of the MOF framework starts in both cases at *ca.* 300 °C and is completed below 400 °C, as evidenced by the second major weight-loss corresponding to the combustion of the organic linker, the associated collapse of the framework and release of volatile species (CO and CO_2_). These results were confirmed by the exothermic peaks at *ca.* 398 and 363 °C, values in accordance with literature.^31,36^ A smaller exothermic event centered at 281 °C is detected for the composite precursor and may be assigned to the decomposition of less thermally stable organic species or framework defects prior to the collapse of the MOF structure. For UiO-66(Ce), the weight loss is of *ca.* 54%, value which agrees with the decomposition of the MOF Ce_6_O_4_(OH)_4_(BDC)_6_ unit cell into CeO_2_. For TiO_2_/UiO-66(Ce), the weight loss is reduced to *ca.* 35%, indicating that the loading in TiO_2_ in TiO_2_/UiO-66(Ce) is of *ca.* 20%. Both TGA profiles show that the obtained CeO_2_ remains stable over the 400–800 °C temperature range, with no further weight changes observed, further confirming the complete thermolysis of the UiO-66(Ce) below 400 °C.

The obtained TiO_2_/UiO-66(Ce) precursor was subsequently calcined under different operating conditions by varying the temperature, the heating rate, and time. The resulting TiO_2_/CeO_2_ materials were evaluated for their adsorption capacity and photoprotective properties toward KP in aqueous solution (Table 1).

**Table 1:** Optimization of TiO_2_/CeO_2_ synthesis conditions and physicochemical characteristics of the obtained materials

Sample	*T* (°C)	Heating rate (°/min)	Time (h)	KP adsorption 1-(*C*_*t*_/*C*_0_)	KP degradation at 240 min (*C*_*t*_*/C*_0_)	Bandgap (eV)
*D*_0_	450	10	2	64.51	89.36	3.05
*D*_1_	600	30	2	39.37	94.51	3.06
*D*_2_	800	30	2	1.86	72.26	3.04
*D*_3_	400	30	2	61.34	59.93	3.07
*D*_4_	800	50	2	10.84	81.91	3.05
*D*_5_	800	30	4	10.09	84.84	3.05

Based on the obtained results, sample *D*_2_ was selected for further studies, as it exhibits the lowest KP adsorption while showing a moderate photocatalytic degradation efficiency. Therefore, *D*_2_ was considered the most suitable material for subsequent mechanistic and performance investigations. In the rest of this study, the TiO_2_/CeO_2_ composite used will correspond to the *D*_2_ sample. In addition, the influence of calcination conditions on the optical properties of the obtained materials was evaluated using diffuse reflectance spectroscopy (DRS) and bandgap estimation using Tauc plots. The DRS spectra of samples *D*_0_–*D*_5_, together with the corresponding Tauc plots are presented in Fig. S1. The obtained results indicate that variations in calcination temperature, heating rate, and dwell time had only moderate impact on the absorption behavior or optical bandgap of the materials.

**Fig. 4:**
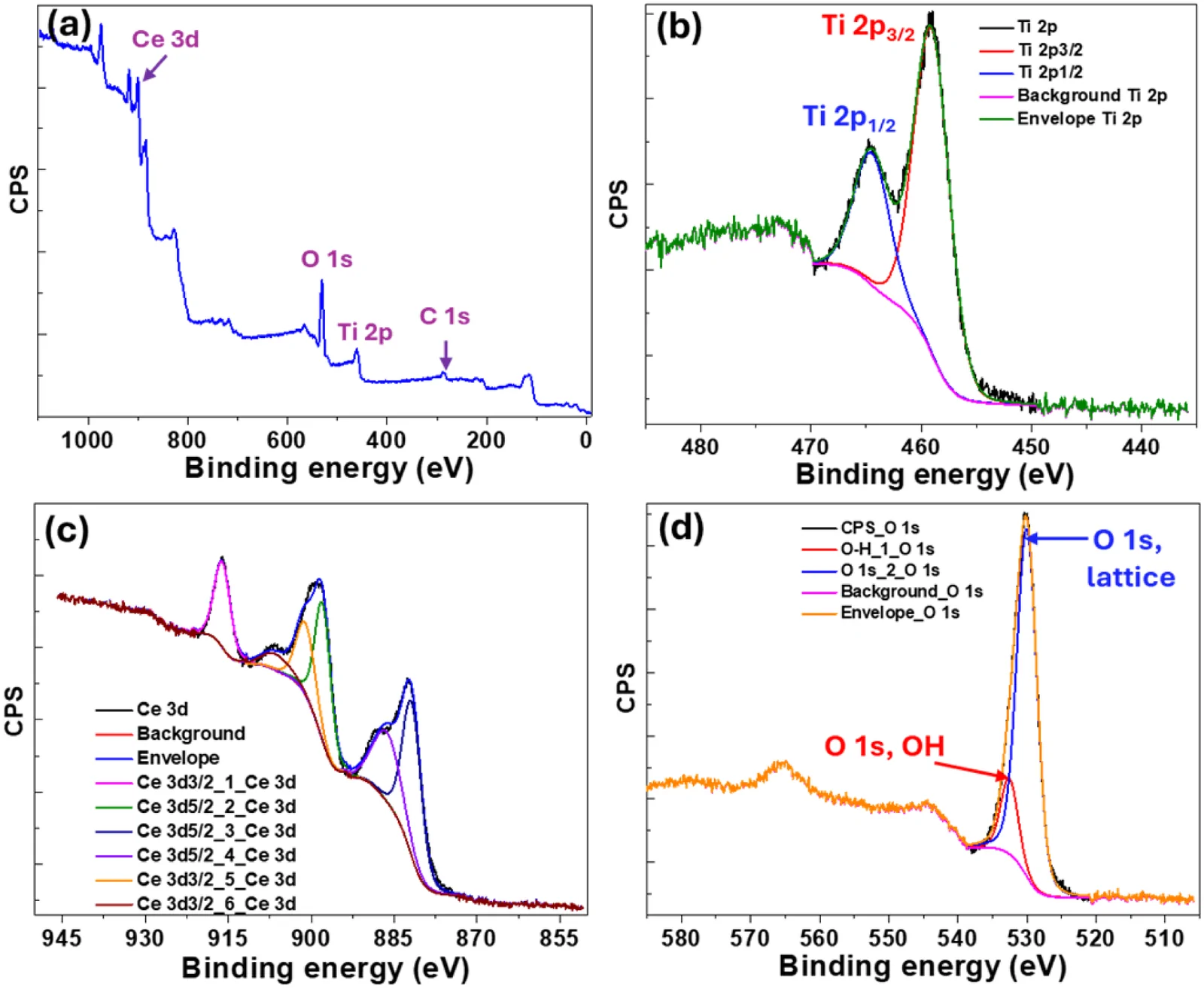
(a) Survey XPS spectrum of TiO_2_/CeO_2_. High resolution XPS spectra of (b) Ti 2p, (c) Ce 3d and (d) O 1s.

The surface composition and the chemical states of elements in the TiO_2_/CeO_2_ composite were analyzed using XPS (Fig. 4). The survey spectrum shows the presence of Ti, Ce and O elements (Fig. 4a). The high resolution XPS spectrum of Ti 2p can be deconvoluted in two signals located at 464.56 and 459.25 eV corresponding to Ti 2p_1/2_ and Ti 2p_3/2_, respectively. The positions of these signals confirm that Ti is in the +4 oxidation state and in a tetragonal structure.^37^ The high resolution XPS spectrum of Ce 3d is more complex and shows six signals located at 916.33, 906.89, 901.25, 898.12, 886.92 and 882.19 eV that can be assigned to the Ce 3d_5/2_ and Ce 3d_3/2_ spin–orbit components, indicating that Ce is in the +4 oxidation state.^38^ These results confirm that Ce^3+^ is oxidized into Ce^4+^ after thermolysis of UiO-66(Ce) under air. For O 1s, the lattice oxygen atoms of TiO_2_ and CeO_2_ can be observed at 530.32 eV, value in accordance with literature.^39^ The O 1s signal observed at 532.31 eV corresponds to surface hydroxyl groups.

Nitrogen adsorption–desorption measurements were performed for UiO-66(Ce), TiO_2_, CeO_2_ (obtained by calcination of UiO-66(Ce) under the conditions used for sample *D*_2_), and TiO_2_/CeO_2_ in order to evaluate their textural properties. The N_2_ adsorption–desorption isotherms and the pore size distributions are presented in Fig. S2 and S3, respectively, and the results are summarized in Table 2. UiO-66(Ce) displays a combined type I/type IV adsorption behavior, confirming the coexistence of micro- and mesopores.^40^ The presence of microporosity was evidenced by a sharp increase in N_2_ uptake at very low relative pressures (*P*/*P*_0_ < 0.02), which is related to pore filling within the UiO-66(Ce) framework.^36^ However, this behavior may not be exclusively associated with microporosity, and contributions from strong adsorbate–adsorbent interactions during monolayer adsorption cannot be excluded. Micropore analysis using the t-plot method yielded a micropore area of 970.2 m^2^ g^−1^, a value slightly lower than those reported in the literature (*ca.* 1280 m^2^ g^−1^ (ref. 34 and 36)). Micropore analysis using the *t*-plot model yields a micropore volume of 0.3 cm^3^ g^−1^, confirming that the majority of UiO-66(Ce) porosity originates from framework micropores. The median micropore width, determined using the Horvath–Kawazoe model^42^ is 5.2 Å. TiO_2_, CeO_2_, and TiO_2_/CeO_2_ samples exhibit type IV isotherms according to the IUPAC classification, indicating the presence of mesopores. All samples exhibited H3-type hysteresis loops between the adsorption and desorption branches,^40^ that can be associated to non-rigid agglomerates of particles and to larger textural voids or macropores.^40,41^ For TiO_2_, CeO_2_ and TiO_2_/CeO_2_, pore-size analysis was performed using the BJH model.^43^ The TiO_2_/CeO_2_ composite exhibits both lower BET surface area and average pore width than rutile TiO_2_ suggesting that the surface of TiO_2_ becomes less accessible after thermal decomposition of the TiO_2_/UiO-66 precursor.

**Table 2:** Textural properties of UiO-66(Ce), CeO_2_, TiO_2_ and TiO_2_/CeO_2_

Sample	*S*_BET_ (m^2^ g^−1^)	Total pore volume (cm^3^ g^−1^)	Average pore width (Å)
UiO-66(Ce)	970.2	0.4	5.2
CeO_2_	25.7	0.1	160.9
TiO_2_	39.1	0.2	141.2
TiO_2_/CeO_2_	24.8	0.1	161.8

**Fig. 5:**
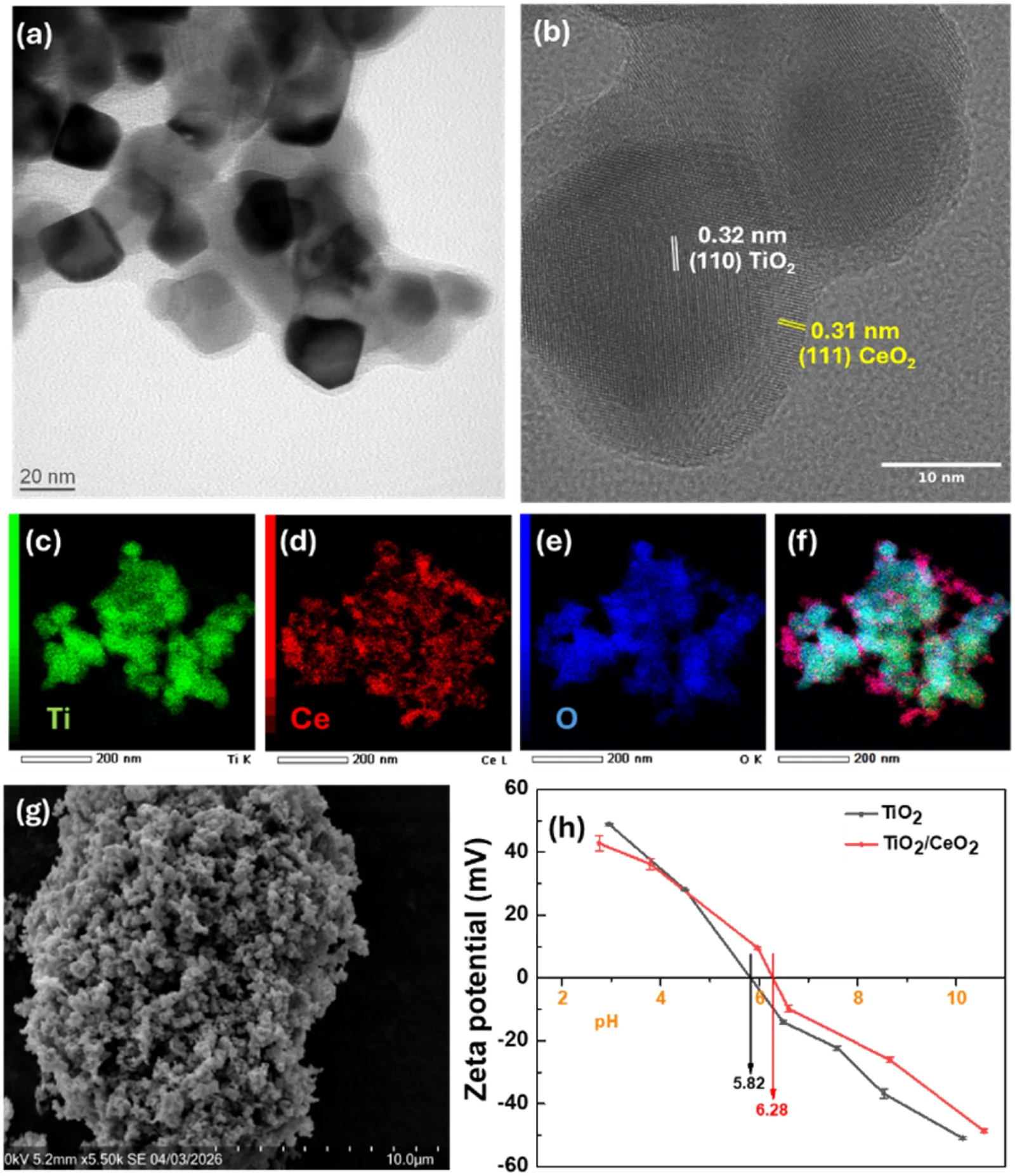
(a and b) HR-TEM images of the TiO_2_/CeO_2_ composite. (c–f) EDX elemental mapping. (g) SEM image of the TiO_2_/CeO_2_ composite. (h) Effect of suspension pH on the Zeta potential of TiO_2_ and TiO_2_/CeO_2_. The isoelectric points were determined as pH 5.82 for TiO_2_ and pH 6.28 for TiO_2_/CeO_2_. Data are presented as mean ± SD (*n* = 3).

TEM analysis of UiO-66(Ce) shows loosely packed aggregates with irregular morphology and a rough and porous texture, which agrees with BET results (Fig. S4). After calcination, the produced CeO_2_ is composed of denser and more compact aggregates, indicating the collapse of the MOF structure (Fig. S5). High resolution TEM images of the TiO_2_/CeO_2_ composite (Fig. 5a and b) show self-associated NPs with marked interfacial contact (TiO_2_ appearing in black and CeO_2_ in grey). The CeO_2_ shell is visible at the edges of TiO_2_, thus confirming the partial surface coverage of TiO_2_ by CeO_2_, which agrees with BET analyses. The distance between the lattice fringes of 0.32 and 0.31 nm can be assigned to the (110) and (111) planes of rutile TiO_2_ and cubic CeO_2_ (Fig. 5b), respectively. The EDX analysis and the associated elemental mapping show the presence of Ti, Ce and O elements (Fig. 5c–f and S6). These results further confirm the coexistence of crystalline TiO_2_ and CeO_2_ phases and the successful formation of a TiO_2_/CeO_2_ composite.

SEM observations reveal that the TiO_2_/CeO_2_ composite is composed of irregular micrometer-sized agglomerates of much smaller primary particles (Fig. 5g). UiO-66(Ce) exhibits a more open, loosely packed morphology, consistent with its large specific surface area (Fig. S7). In contrast, the TiO_2_/CeO_2_ sample shows denser and more compact agglomerates, confirming the particle sintering during calcination of the TiO_2_/UiO-66(Ce) composite. EDX analyses were conducted to determine the elemental composition of the materials before and after calcination (Table S2). The UiO-66(Ce) sample contains carbon and oxygen originating from the BDC linker and inorganic cluster nodes, together with Ce. After encapsulation of TiO_2_, the TiO_2_/UiO-66(Ce) composite shows the presence of titanium. Following calcination, the TiO_2_/CeO_2_ material contained only Ti, Ce, and O, confirming the effective conversion of the precursor into oxides.

The surface charge of the materials was evaluated by measuring the Zeta potential as a function of pH which is of high importance for adsorption. When considering topical applications, it is desirable to minimize electrostatic attraction within the pH range relevant for dermal formulations. There is broad agreement that skin-applied products should be mildly acidic, typically within pH 4–6.^44^ As can be seen in Fig. 5h, both TiO_2_ and TiO_2_/CeO_2_ show pH-dependent behavior, with positive Zeta potential values under acidic conditions and negative values at alkaline pH. The isoelectric point (IEP) of rutile TiO_2_ is 5.82, whereas the TiO_2_/CeO_2_ composite showed a shifted IEP of 6.28.

The Zeta potential results also explain the low KP adsorption observed for the TiO_2_/CeO_2_ sample. The pH of the KP solution (20 mg L^−1^) was 4.31 at 25 °C, while the experimentally determined p*K*_a_ of KP was 4.45.^45^ According to the Henderson–Hasselbalch equation, approximately 42% of KP was present in the ionized form and 58% in the neutral form under these conditions, indicating that non-ionized species predominated. At this pH value, the TiO_2_/CeO_2_ surface exhibits a strongly positive Zeta potential of *ca.* +29 mV, which could favor adsorption of the anionic species. However, despite this electrostatic interaction, the overall adsorption remained low, indicating that the limited specific surface area and the reduced number of accessible adsorption sites are the dominant factors controlling KP uptake.

**Fig. 6:**
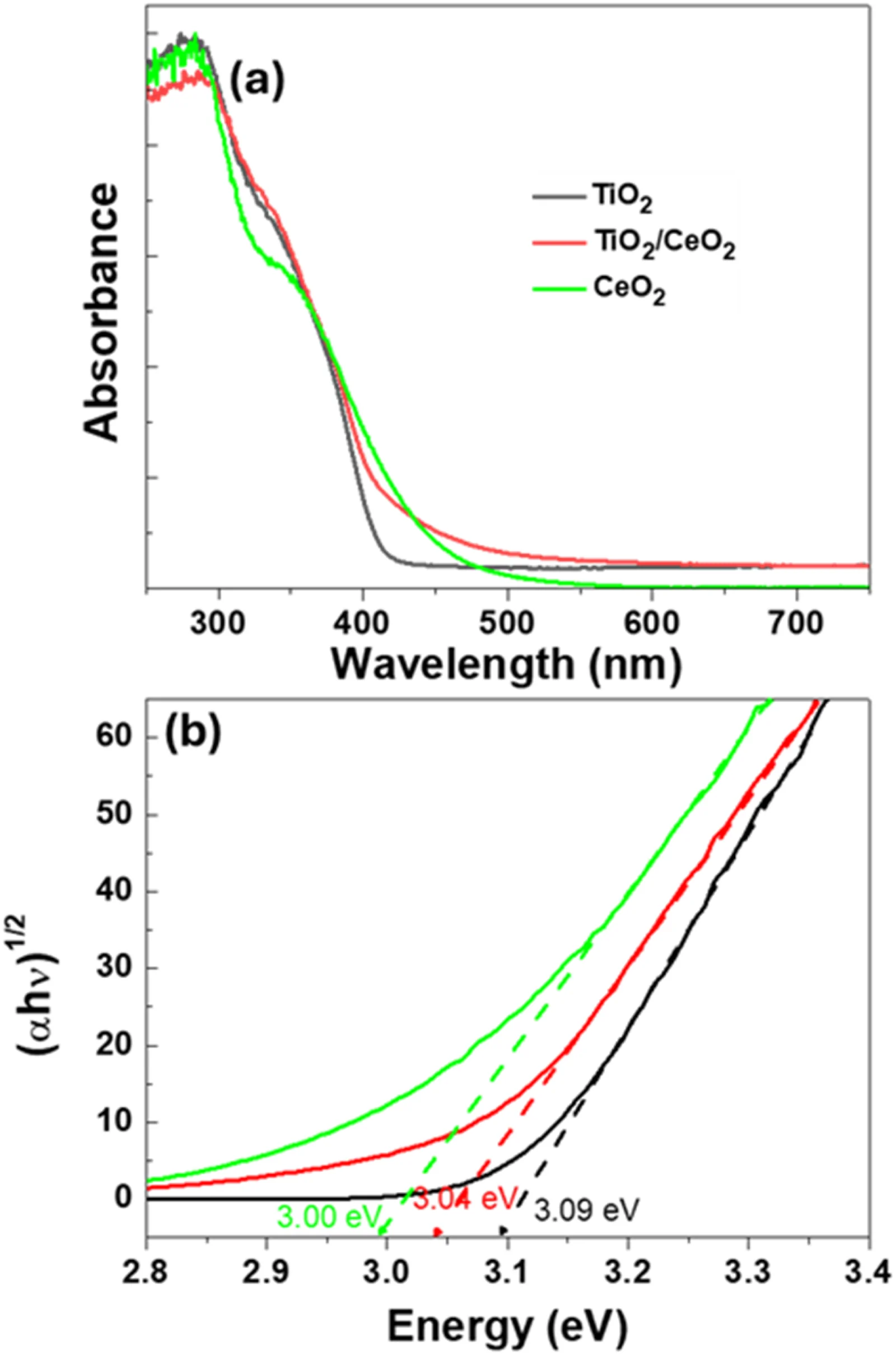
UV-vis diffuse reflectance spectra (a) of TiO_2_, CeO_2_, and TiO_2_/CeO_2_ and (b) the corresponding Tauc plots used for estimation of the optical band gap energy.

Diffuse reflectance spectroscopy (DRS) was performed to evaluate the optical properties of TiO_2_, CeO_2_, and TiO_2_/CeO_2_ composite (Fig. 6). All samples exhibit strong absorption in the UV region which is characteristic of wide bandgap metal oxides. Compared to TiO_2_, the TiO_2_/CeO_2_ heterostructure shows a noticeable redshift of the absorption edge, indicating changes in the optical response and further improvement in the UV-shielding ability. This behavior is consistent with literature reports describing the extended CeO_2_ light absorption in the visible range.^46^ Bandgap energies estimated from Tauc plots were consistent with these observations, confirming that the TiO_2_/CeO_2_ material retained the wide-bandgap character of the parent oxides while exhibiting a redshifted absorption onset.

## ROS photoproduction

### Superoxide radicals (O_2_˙^−^) generation

**Fig. 7:**
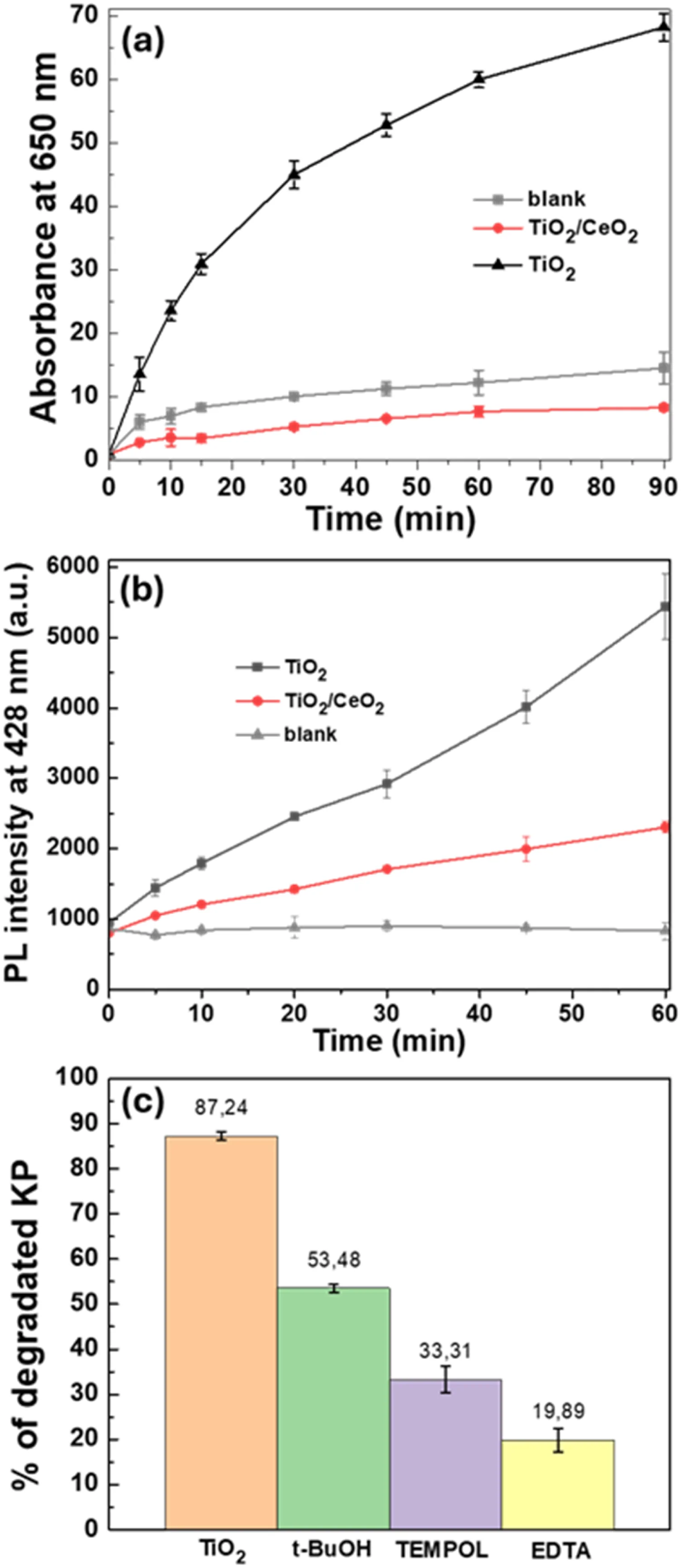
(a) Generation of O_2_˙^−^ evaluated by the NBT assay during UVA irradiation of TiO_2_ and TiO_2_/CeO_2_ dispersions. Results are presented as the increase in absorbance at 650 nm corresponding to the formation of formazan products. Blank represents the irradiated NBT solution in the absence of catalyst. Data are shown as mean ± SD (*n* = 3). (b) Generation of ˙OH radicals evaluated by the DST assay during UVA irradiation of TiO_2_ and TiO_2_/CeO_2_ suspensions. Results are expressed as PL intensity at 428 nm corresponding to the formation of 2-hydroxyterephthalate. Blank represents the irradiated DST solution without a catalyst. Data are presented as mean ± SD (*n* = 3). (c) Reactive species scavenging experiments during TiO_2_-mediated photocatalytic degradation under UVA irradiation of KP. *Tert*-Butanol, TEMPOL and EDTA were used as scavengers for ˙OH, O_2_˙^−^ and h^+^, ^1^respectively. Data are presented as mean ± SD (*n* = 3).

The ability of the materials to generate O_2_˙^−^ radicals under UVA irradiation was evaluated using the NBT assay (Fig. 7a). In this method, the reduction of NBT to formazan derivatives is monitored spectrophotometrically by the increase in absorbance at 650 nm. As expected, using TiO_2_, a fast and continuous increase in absorbance is observed, indicating the efficient generation of O_2_˙^−^ radicals under UVA irradiation. This observation is consistent with the well-established photocatalytic activity of TiO_2_ for which photogenerated electrons in the conduction band reduce dissolved oxygen to O_2_˙^−^ species.^47^ In contrast, a weak increase in absorption at 650 nm is observed for the TiO_2_/CeO_2_ composite, indicating that the association of TiO_2_ with CeO_2_ almost fully inhibits the production of O_2_˙^−^ radicals. Moreover, the intensity of the absorption is lower than the blank experiment, suggesting that CeO_2_ may exhibit O_2_˙^−^-scavenging or antioxidant behavior, which agrees with the superoxide dismutase mimetic activity of CeO_2_ NPs.^48^ The marked reduction in O_2_˙^−^ production for the TiO_2_/CeO_2_ sample is consistent with its physicochemical characterizations. The lower specific surface area of TiO_2_/CeO_2_ compared to TiO_2_ reduces the number of accessible photoactive sites, while partial surface coverage of TiO_2_ by CeO_2_ may hinder direct interfacial electron transfer to molecular oxygen. In addition, the close contact between TiO_2_ and CeO_2_ may promote charge recombination or electron trapping within CeO_2_. These results are of interest from the perspective of topical and dermal applications, as O_2_˙^−^ radicals are ROS involved in oxidative degradation of APIs and may contribute to phototoxic side effects on the skin.^49^

### Hydroxyl radical (˙OH) generation

The generation of ˙OH radicals under UVA irradiation was investigated using the DST fluorescence assay (Fig. 7b). In this method, ˙OH radicals react with DST to form fluorescent 2-hydroxyterephthalate and the increase in emission intensity at 428 nm is directly related to hydroxyl radical production.

As shown in Fig. 7b, no fluorescence is observed when irradiating DST, indicating that the photoproduct did not form in the absence of photocatalytically active materials. By contrast, a strong and continuous increase in fluorescence intensity is observed for pure TiO_2_, indicating efficient generation of ˙OH radicals upon UVA exposure *via* oxidation of water by photogenerated valence band holes.^16^ The TiO_2_/CeO_2_ composite also generates ˙OH radicals but a much lesser extent. After 60 min of irradiation, the fluorescence intensity is reduced by *ca.* 58% compared to TiO_2_. This effect may be attributed to several complementary factors: (i) the composite's lower specific surface area reduces the number of accessible active sites for ROS generation, (ii) the partial coverage of the TiO_2_ surface by the CeO_2_ may block direct hole transfer to adsorbed water molecules and (iii) the redox active Ce^4+^/Ce^3+^ couple can act as a charge-buffering system, facilitating the recombination or trapping of photogenerated charge carriers before radical formation. From an application standpoint, this observation is highly relevant because ˙OH radicals are among the most aggressive ROS and can initiate oxidative degradation of active pharmaceutical ingredients, excipients, and biological components in topical formulations or in skin tissue.^50,51^

For KP (Fig. 7c), the photocatalytic degradation using TiO_2_ in the absence of scavengers reached 87.24%. The addition of *tert*-butanol reduced degradation to 53.48%, indicating that ˙OH radicals contributed to the process. A stronger inhibition was observed upon addition of TEMPOL and EDTA, which reduced degradation to 33.31 and 19.89%, respectively, demonstrating that O_2_˙^−^ radicals and photogenerated h^+^ were the dominant reactive species responsible for KP degradation, whereas ˙OH radicals played a secondary role. Thus, the contribution of reactive species followed the order: h^+^ > O_2_˙^−^ > ˙OH. This suggests that KP degradation occurred *via* a combined mechanism involving direct oxidation by valence band holes and indirect oxidation by ROS generated on the TiO_2_ surface. These findings are consistent with previous reports showing that O_2_˙^−^ radicals are major contributors to KP photodegradation.^49^ The significant contribution of photogenerated holes observed here further indicates the importance of direct surface oxidation pathways, as well as their indirect role in facilitating O_2_˙^−^ generation using TiO_2_.

The reduced generation of both O_2_˙^−^ and ˙OH radicals observed in the present study is consistent with previous reports. Morlando *et al.* demonstrated that decorating TiO_2_ with CeO_2_ substantially decreased photocatalytic activity while preserving UV attenuation properties and improving biocompatibility.^48^ Similarly, the association of CeO_2_ with TiO_2_ has been shown to reduce the photodegradation of organic dyes and to mitigate UV-induced toxicity by limiting ROS production.^52^ More recently, TiO_2_/Ce_2_O_3_ heterojunctions were engineered to promote charge-carrier recombination, resulting in a marked decrease of the photocatalytic activity and improved suitability for UV-protective applications.^27^ However, these studies primarily focused on model systems such as organic dyes. In contrast, the present work extends this concept to pharmaceutical applications by directly demonstrating that ROS suppression translates into efficient photoprotection of a photosensitive active pharmaceutical ingredient.

### Photodegradation of KP in hydrogel

**Fig. 8:**
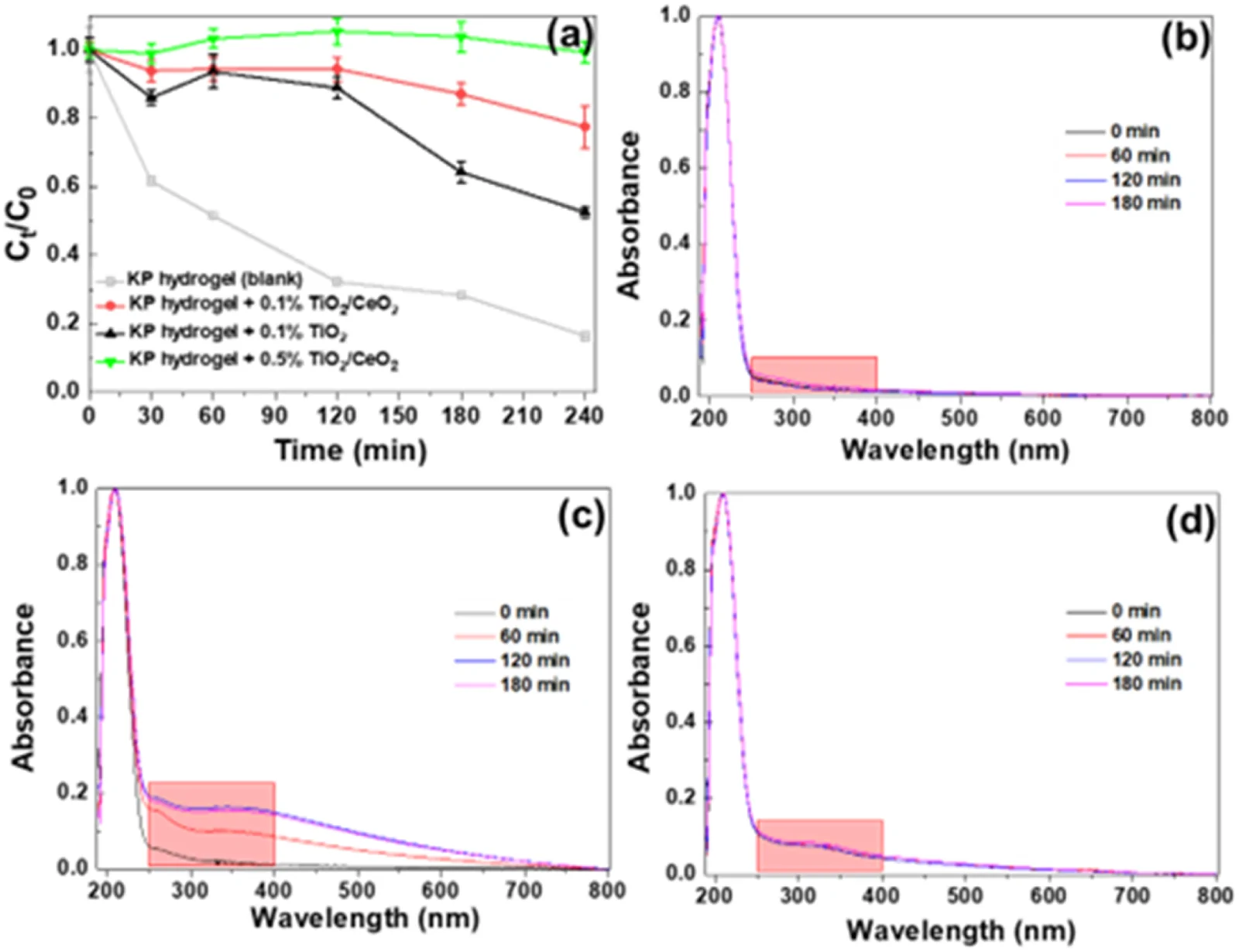
(a) Photodegradation of ketoprofen (KP) in hydrogel formulations under UVA irradiation in the presence of the investigated materials. Results are expressed as the normalized concentration ratio (*C*_*t*_/*C*_0_). Data are presented as mean ± SD (*n* = 3). UV-vis absorption spectra of extracts obtained from carbopol-based hydrogels after UVA irradiation: (b) blank hydrogel, (c) hydrogel containing 0.1% TiO_2_, and (d) hydrogel containing 0.1% TiO_2_/CeO_2_.

The photostability of KP was further evaluated in hydrogel formulations containing the investigated materials under UVA irradiation (Fig. 8a). In contrast to aqueous solution, this experiment was designed to better reflect practical semisolid dosage forms used for topical administration. In the pure hydrogel, a pronounced KP degradation is observed with only 16.44% of the initial drug remaining after 240 min of irradiation indicating the poor photostability of the drug. Incorporation of 0.1% TiO_2_ in the hydrogel improved the photostability and the remaining KP content is 52.56%. An increased KP photostability is observed for the 0.1% TiO_2_/CeO_2_ with 77.54% of KP remaining after the same irradiation period. To determine the minimum material content required to achieve complete photoprotection, the composite loading was increased. A complete protection is observed with the formulation containing 0.5% TiO_2_/CeO_2_ and the KP concentration remained essentially unchanged (99.29% remaining) after irradiation. The calculated photostability enhancement factor (PEF) further confirms the composite material's superior protective performance. The PEF values are 1.76 for 0.1% TiO_2_, 3.72 for 0.1% TiO_2_/CeO_2_, and 117.92 for 0.5% TiO_2_/CeO_2_. These results demonstrate a clear concentration-dependent protective effect of the TiO_2_/CeO_2_ composite and indicate that CeO_2_ significantly improves the ability of TiO_2_/CeO_2_ composite to stabilize photosensitive active ingredients in hydrogel formulations under UVA exposure. This concentration-dependent effect is particularly important considering thatTiO_2_ is typically used in external-use formulations for its protective and covering properties at concentrations ranging from 1 to 10%. Therefore, the effective composite loadings applied in the present study remain well within the range commonly used in topical products.

Our findings can be discussed in the context of an earlier study on KP photostabilization in topical systems. Loden *et al.* investigated KP gels containing different grades of TiO_2_ microparticles and demonstrated that the protective effect strongly depended on particle quality and surface treatment. In their *in vitro* model, unprotected gel formulations showed almost complete KP loss after irradiation, whereas the addition of 4% surface-coated TiO_2_ substantially improved KP stability. Specifically, for gels containing an initial KP concentration of 2.5%, approximately 0.7% KP remained after sunlight exposure, while about 0.4% remained after irradiation using a solar simulator, demonstrating significant, although incomplete, photoprotection. In contrast, highly photoactive fine-crystalline TiO_2_ provided little or no protection, confirming that UV shielding alone is insufficient when photocatalytic activity is not adequately controlled.^12^ Although direct numerical comparison should be made cautiously due to major differences in formulation composition, particle size (microparticles *vs.* nanoparticles), irradiation source, dose, exposure geometry, and analytical protocol, the overall conclusion is highly consistent with the present work: effective photostabilization requires a balance between optical UV attenuation and minimized photoinduced reactivity.

To further examine the influence of the investigated materials on the hydrogel matrix, UV-vis spectra were recorded from irradiated hydrogels prepared without KP (Fig. 8b–d). This experiment is intended to assess whether the presence of the inorganic additives affected the photostability of the polymeric vehicle under UVA exposure. For the blank experiment (Fig. 8b), the UV-vis absorption spectra remained almost unchanged throughout the irradiation period, with only a slight increase in absorbance after 180 min. A very similar behavior is observed for the hydrogel containing 0.1% TiO_2_/CeO_2_ (Fig. 8d), indicating that the composite did not induce pronounced alterations in the matrix under the applied conditions. In contrast, more pronounced spectral changes are observed for the hydrogel containing 0.1% TiO_2_ (Fig. 8c). In this case, a clear increase in absorbance was observed during irradiation over a broad wavelength range, suggesting the formation of new UV-vis absorbing species or structural modifications within the hydrogel matrix. Since KP was absent from these samples, the observed effect must be attributed to interactions between photoactivated TiO_2_ and the formulation components. The hydrogel was Carbopol, a hydrophobically modified highly crosslinked acrylic acid copolymer, and the obtained results suggest that TiO_2_ photocatalytic activity may promote chemical or physicochemical changes within this polymer network during UVA exposure. Although the exact nature of these transformations was not identified in the present study, the data clearly indicate that unmodified TiO_2_ affects the matrix stability to a much greater extent than the TiO_2_/CeO_2_ composite. A plausible explanation is that ROS generated during irradiation reacted with the polymer chains, leading to oxidative degradation and the formation of new products containing chromophores. This observation further supports the beneficial effect of CeO_2_ in reducing undesired photoinduced reactivity not only toward active ingredients, but also toward the formulation vehicle itself.

### Ecotoxicity assessment – microtox acute toxicity study

**Fig. 9:**
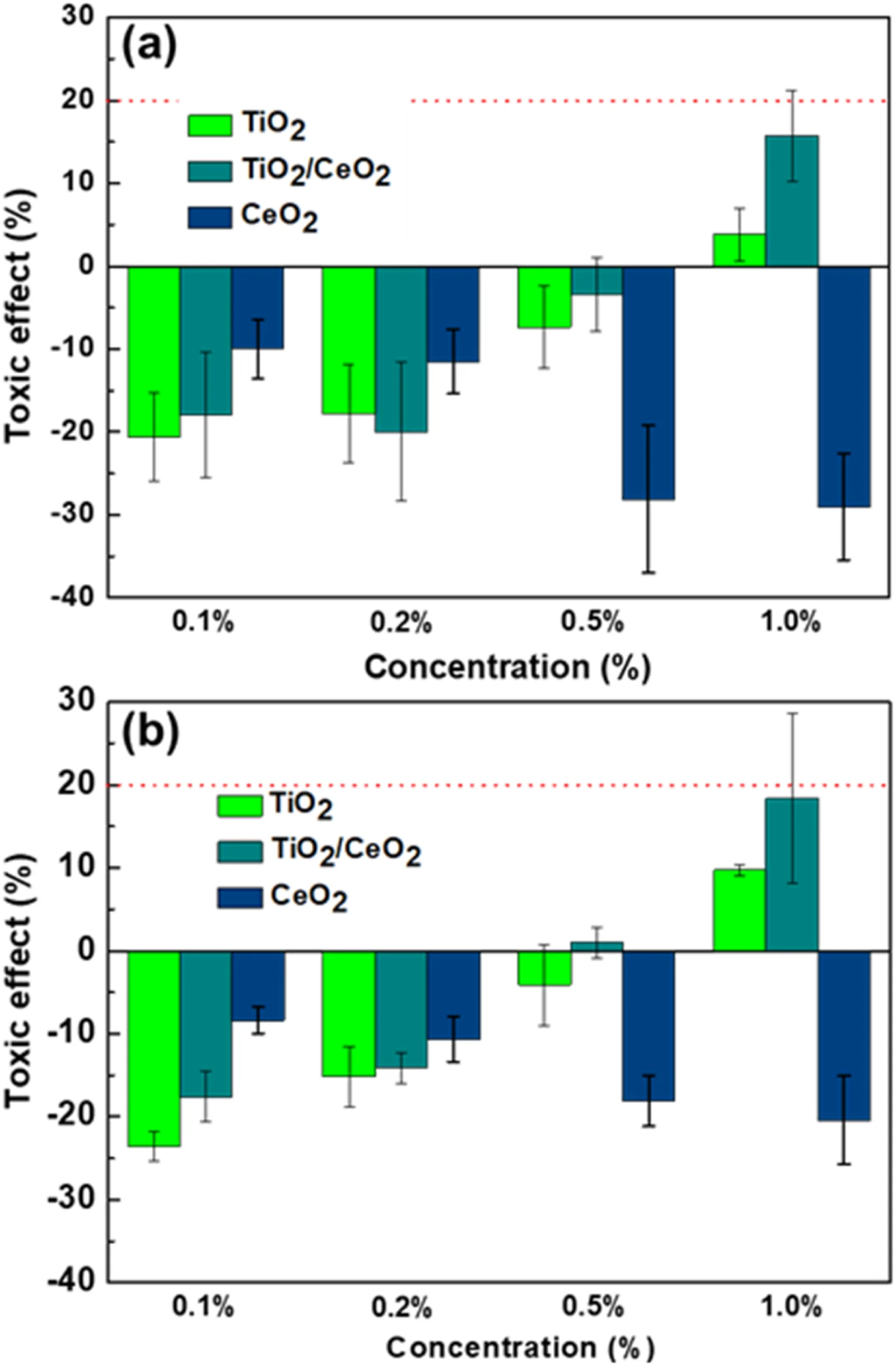
Acute toxicity of TiO_2_, TiO_2_/CeO_2_, and CeO_2_ suspensions determined by the Microtox assay after (a) 5 min and (b) 15 min of exposure. Results are expressed as toxic effect (%). Data are presented as mean ± SD (*n* = 3).

The acute toxicity of the investigated materials toward *Aliivibrio fischeri* was evaluated after 5 min (Fig. 9a) and 15 min (Fig. 9b) of exposure. According to the accepted interpretation of the Microtox screening assay, samples causing ≤20% inhibition are considered non-toxic,^53^ while negative values, corresponding to an increase in bioluminescence, may indicate a hormetic-type stimulatory response.^54^

Overall, all tested materials show low acute toxicity within the investigated concentration range (0.1–1.0%), since none of the measured effects exceeds the 20% threshold. In most cases, negative values are observed, indicating no measurable harmful effect on bacterial bioluminescence and potentially stimulation of metabolic activity. At lower concentrations (0.1–0.5%), all materials produced negative responses for both exposure times.

The most pronounced response is observed at 1.0%, where TiO_2_ and especially TiO_2_/CeO_2_ produce positive inhibition values. For the TiO_2_/CeO_2_ composite, the effect reached the 20% threshold after 15 min, suggesting that toxicity may emerge only at the highest tested concentration. Nevertheless, the measured value remains below the classification threshold for toxicity. In contrast, CeO_2_ maintains negative values even at 1.0%, indicating no acute toxic response in this assay, consistent with the literature.^55^ Titania NPs did not exert a significant toxic effect.^56^

Comparison of the 5- and 15-min results shows only minor time-dependent changes, suggesting that the tested suspensions do not induce strong delayed toxicity. Taken together, these findings indicate that the TiO_2_/CeO_2_ composite retained a favorable acute ecotoxicological profile, particularly at concentrations relevant to the formulation studies (0.1–0.5%).

Despite the promising results obtained in this study, several limitations should be considered. First, the TiO_2_/CeO_2_ composite was evaluated under controlled laboratory conditions using a monochromatic UVA source and simplified model environments. Although this approach enables reliable mechanistic comparisons, it does not fully reproduce real sunlight exposure, which is more complex in terms of spectral distribution and irradiation conditions. Second, the functional performance of the material was assessed using selected model systems, namely CV, KP, and hydrogel formulations. While these models are relevant and informative, broader validation using additional photosensitive active ingredients and more complex dosage forms is still required. Third, although the TiO_2_/CeO_2_ composite demonstrated reduced ROS generation and favorable Microtox results, its safety profile remains incomplete and requires further investigation before practical pharmaceutical or dermal application.

Future studies will therefore focus on a comprehensive toxicological evaluation of the developed material, including its effects on 3D skin models, phototoxicity, influence on the immune response, and potential NPs penetration through the skin barrier. In parallel, the applicability of the TiO_2_/CeO_2_ system will be extended to the photoprotection of other photosensitive drugs and to more advanced topical formulations.

## Conclusions

In this work, heterostructured TiO_2_/CeO_2_ NPs were developed to overcome one of the key limitations of TiO_2_ in topical applications, primarily its undesirable photocatalytic reactivity under UV exposure. The applied UiO-66(Ce)-derived synthesis route enabled the formation of a closely contacted oxide heterostructure in which CeO_2_ effectively modulated the physicochemical and functional behavior of TiO_2_. Results obtained demonstrate that the incorporation of CeO_2_ significantly reduces ROS generation under UVA irradiation, particularly superoxide radicals. At the same time, the composite retained the beneficial UV-screening properties of TiO_2_, thereby enhancing KP protection against photodegradation. This effect was especially evident in hydrogel formulations, where the TiO_2_/CeO_2_ composite outperformed TiO_2_, achieving complete KP photoprotection at only 0.5 weight% loading. Importantly, the association of TiO_2_ with CeO_2_ also reduces undesirable photoinduced effects toward the hydrogel vehicle itself, indicating that the benefits of the developed system extend beyond API stabilization to improved formulation compatibility. Furthermore, the favorable Microtox profile suggests that the material exhibits low acute ecotoxicity over the concentration range relevant to formulation use. Overall, the study demonstrates that heterostructuring TiO_2_ with CeO_2_ offers a promising strategy for designing next-generation inorganic excipients with a more favorable balance between UV protection and safety. The developed platform may be particularly valuable for stabilizing photosensitive drugs in dermal and topical dosage forms and provides a strong foundation for future translational and toxicological investigations.

## Author contributions

Michał Gackowski: conceptualization, funding acquisition, investigation, methodology, project administration, validation, visualization, writing – original draft. Dariusz T. Mlynarczyk: investigation, writing – review and editing. Halima Alem: investigation, writing – review and editing. Joanna Budna-Tukan: conceptualization. Tomasz Osmałek: funding acquisition, supervision, writing – review and editing. Raphaël Schneider: conceptualization, funding acquisition, methodology, project administration, resources, supervision, visualization, writing – original draft, writing – review and editing.

## Conflicts of interest

The authors declare that they have no known competing financial interests or personal relationships that could have influenced the work reported in this paper. The authors have no conflicts of interest to declare. All co-authors have seen and agree with the contents of the manuscript.

## Supplementary Material

RA-016-D6RA04759A-s001

## Data Availability

The data supporting this article have been included as part of the supplementary information (SI). Supplementary information: composition of hydrogel formulations, effect of calcination conditions on adsorption and photocatalytic degradation performance, diffuse reflectance UV-vis absorption spectra and Tauc plots, textural parameters from N_2_ adsorption–desorption measurements, HRTEM and SEM images, and EDX elemental composition analysis of UiO-66(Ce), TiO_2_/UiO-66(Ce), and TiO_2_/CeO_2_ materials. See DOI: https://doi.org/10.1039/d6ra04759a.
