# Supplementary material for: Tuning photoactivity and photoprotection of TiO2via TiO2/CeO2 heterostructure composite engineering
Source: RSC Adv. 2026 Jul 28. Online ahead of print. doi: 10.1039/d6ra04759a (PMC13411312; doi:10.1039/d6ra04759a)
Supplement: RA-OLF-D6RA04759A-s001 [file RA-OLF-D6RA04759A-s001.pdf]

Supplementary Information (SI) for RSC Advances.
This journal is © The Royal Society of Chemistry 2026

Electronic Supporting Information

Tuning Photoactivity and Photoprotection of TiO_2 via TiO_2/CeO_2 Heterostructured Composite Engineering

Michał Gackowski,[a,b,c] Dariusz T. Mlynarczyk,[d] Halima Halem,[e] Joanna Budna-Tukan,[f,g] Tomasz Osmałek,[b] and Raphaël Schneider*[a]

[a]Université de Lorraine, CNRS, LRGP, F-54000 Nancy, France. raphael.schneider@univ-lorraine.fr

[b]Poznan University of Medical Sciences, Chair and Department of Pharmaceutical Technology, 3 Rokietnicka Street, 60-806 Poznan, Poland.

[c]Poznan University of Medical Sciences, Doctoral School, 70 Bukowska Street, 60-812 Poznan, Poland.

[d]Poznan University of Medical Sciences, Chair and Department of Chemical Technology of Drugs, Rokietnicka 3, 60-806 Poznań, Poland.

[e]Université de Lorraine, CNRS, IJL, F-54000 Nancy, France.

[f]Poznan University of Medical Sciences, Department of Immunology, 60-806 Poznań, Poland

[g]Department of Anatomy and Histology, Collegium Medicum, University of Zielona Gora, 65-046 Zielona Gora, Poland

Table S1. Composition of the investigated hydrogel formulations. The parameter x denotes the variable amount of TiO_2 or TiO_2/CeO_2 incorporated into the hydrogel, depending on the tested formulation. The water content (H_2O) was adjusted accordingly (6.674 − x) to maintain a constant total composition.

Ingredient	Blank hydrogel	Hydrogel with material
KP	0.1	0.1
Ethanol (absolute)	3.0	3.0
Carbopol® EZ-3	0.2	0.2
Triisopropanolamine (TIPA)	0.026	0.026
TiO_2 or TiO_2/CeO_2	-	x
H_2O	6.674	6.674-x

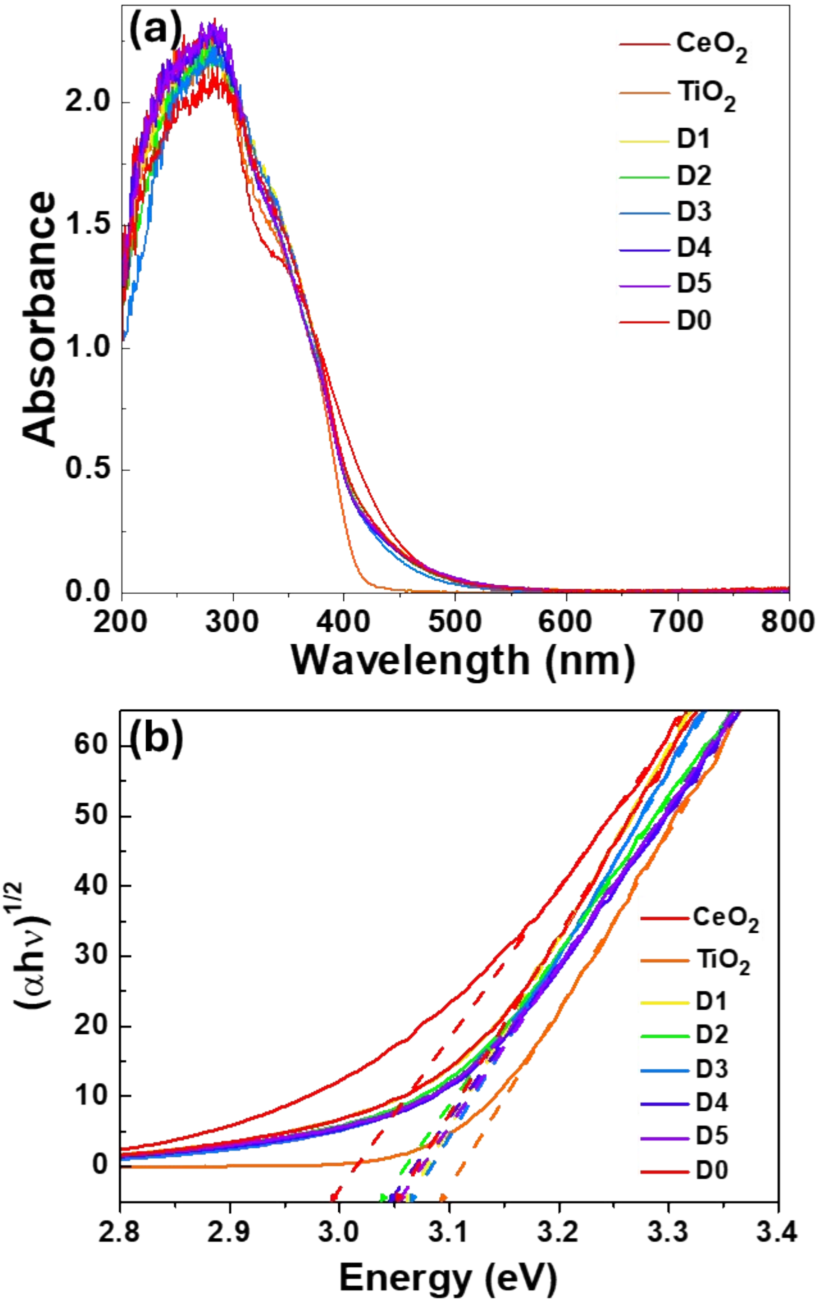

Fig. S1. (a) Diffuse reflectance UV–Vis absorption spectra (DRS) of reference CeO_2, TiO_2, and TiO_2/CeO_2 samples D_0–D_5 obtained under different calcination conditions. (b) Corresponding Tauc plots used for estimation of the optical bandgap energies. The results indicate only minor differences in light absorption behavior and bandgap values among the investigated samples, suggesting that the applied calcination conditions had no significant effect on the optical properties of the resulting materials.

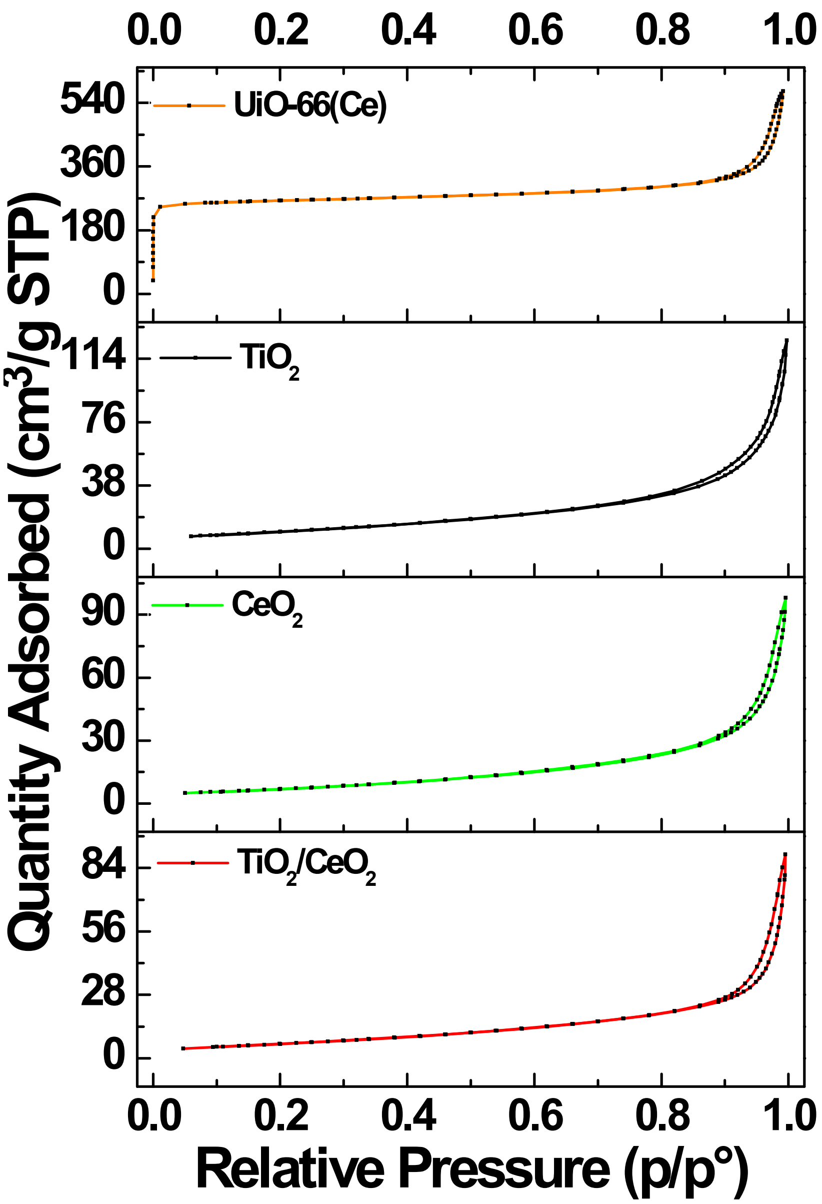

Fig. S2. N_2 adsorption-desorption measurements.

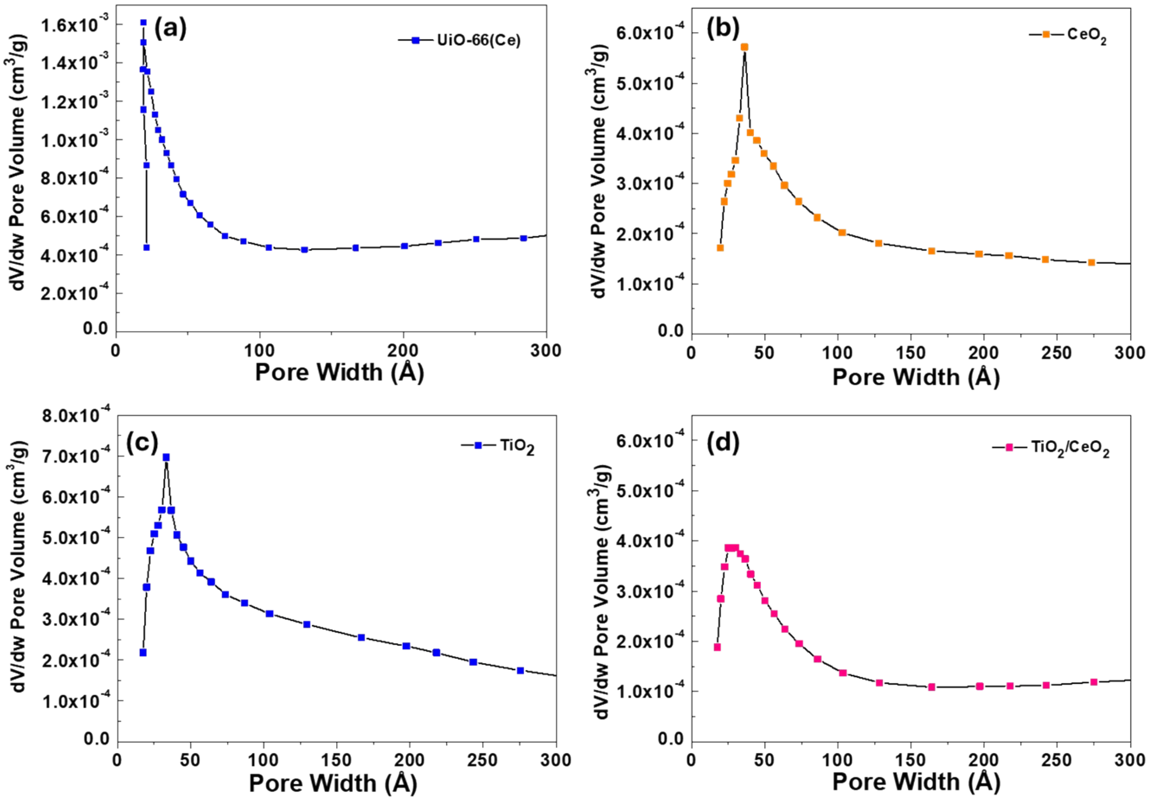

Fig. S3. Pore size distribution of (a) UiO-66(Ce), (b) CeO_2, (c) TiO_2 and (d) TiO_2/CeO_2.

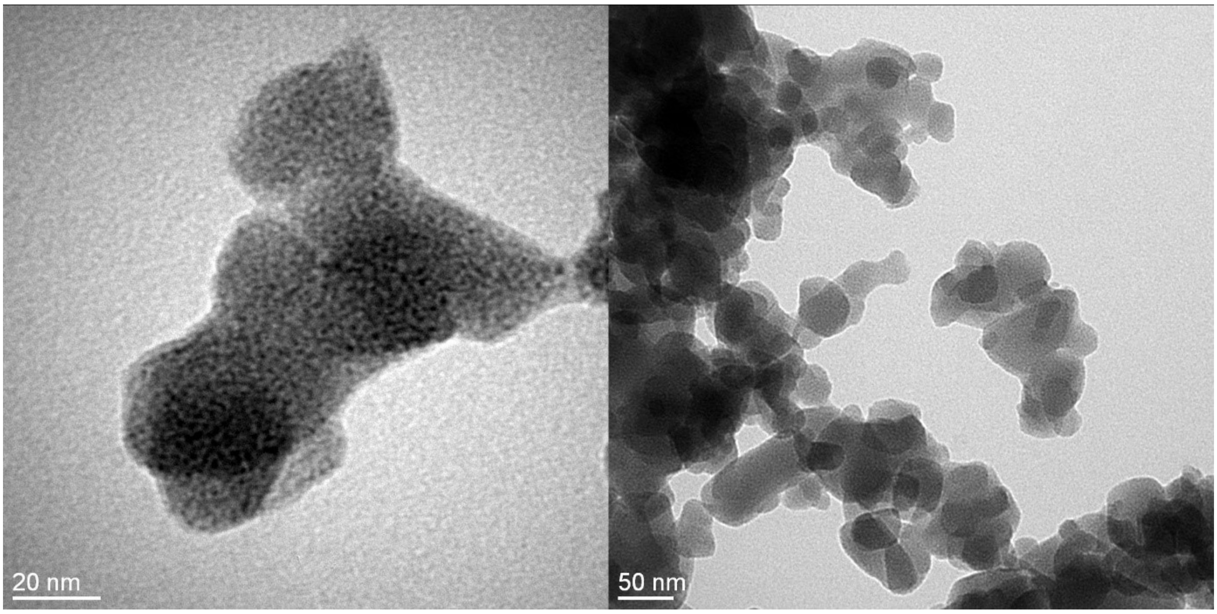

Fig. S4. TEM images of UiO-66(Ce).

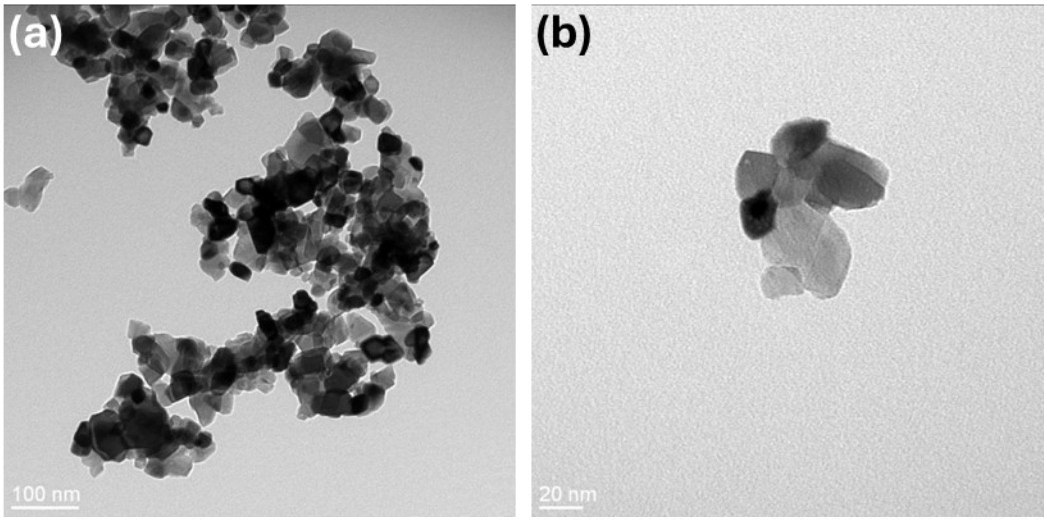

Fig. S5. TEM images of CeO_2 produced by thermolysis of UiO-66(Ce).

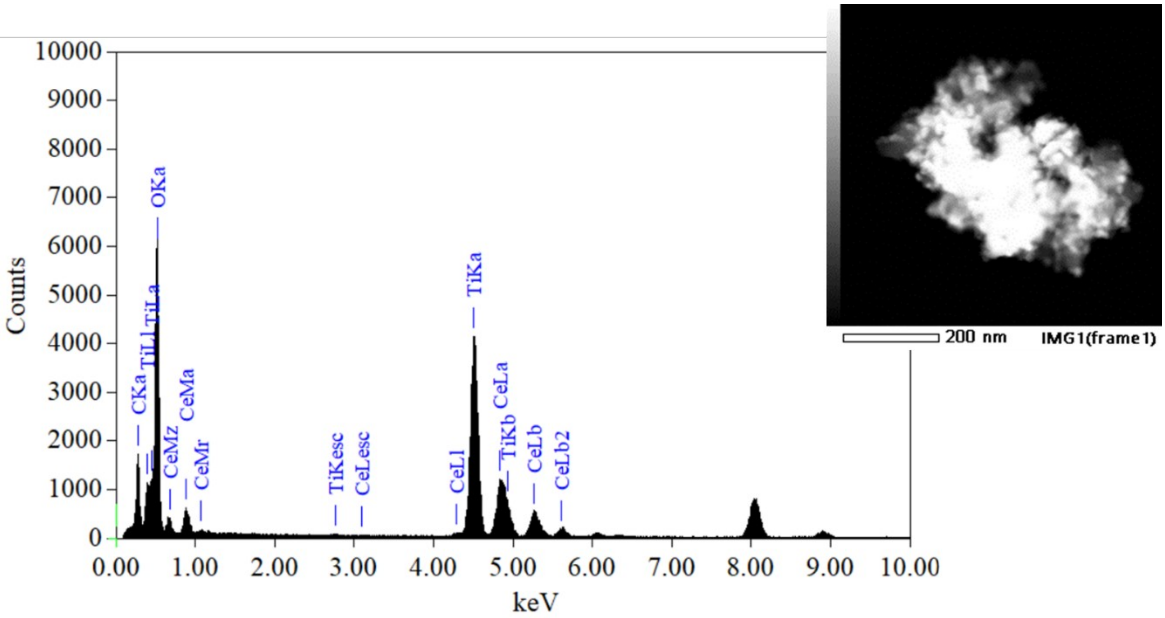

Fig. S6. EDX analysis of the TiO_2/CeO_2 composite.

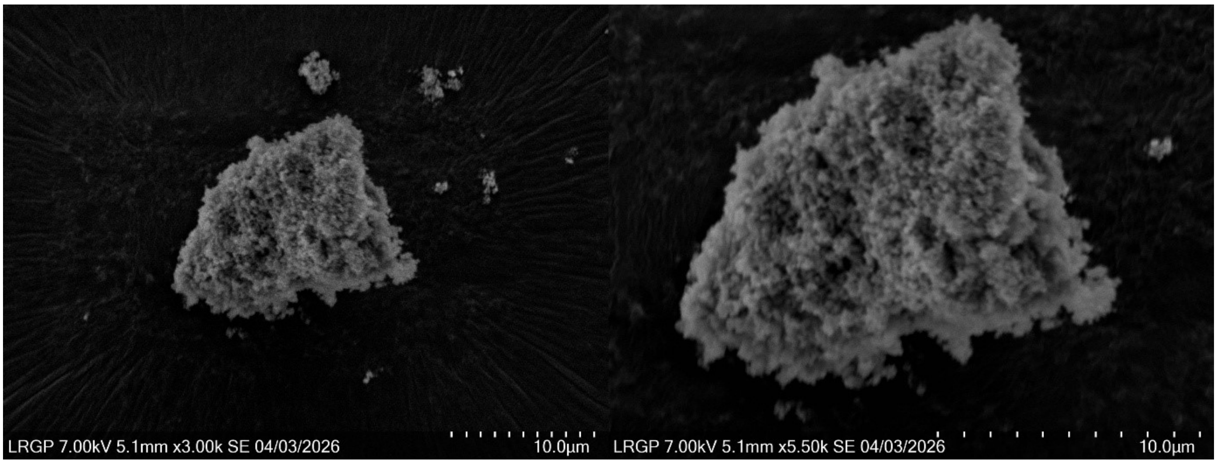

Fig. S7. SEM images of UiO-66(Ce).

Table S2. Elemental composition of UiO-66(Ce), TiO_2/UiO-66(Ce), and TiO_2/CeO_2 determined by EDX and expressed as weight percentage (mean ± SD, n = 3).

**Element**	**Weight [%]**		
	UiO-66(Ce)	TiO_2/UiO-66(Ce)	TiO_2/CeO_2
C	75.87 ± 3.51	54.57 ± 2.13	-
O	19.32 ± 3.03	28.99 ± 1.75	45.59 ± 1.74
Ce	4.81 ± 0.74	10.09 ± 0.60	32.13 ± 1.51
Ti	-	6.35 ± 0.20	22.29 ± 0.23
